# One-Pot Telescoping *S*-Transfer and Trifluoromethylation for the Synthesis of 2-CF_3_S-Imidazoles with *N*-Oxides as Convenient Precursors

**DOI:** 10.1021/acs.joc.4c01761

**Published:** 2024-09-30

**Authors:** Wiktor K. Poper, Jun-An Ma, Marcin Jasiński

**Affiliations:** † Faculty of Chemistry, University of Lodz, Tamka 12, Łódź 91403, Poland; ‡ Department of Chemistry, Tianjin Key Laboratory of Molecular Optoelectronic Sciences, Frontiers Science Center for Synthetic Biology (Ministry of Education), Tianjin University, Tianjin 300072, People’s Republic of China

## Abstract

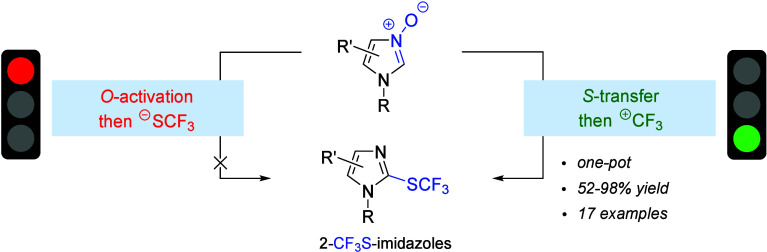

Readily available 2-unsubstituted imidazole *N*-oxides were examined as starting materials for the preparation of fully substituted 1,4,5-aryl/alkyl 2-trifluoromethylsulfanyl-imidazoles. Whereas activation of the *N*-oxide function followed by attempted nucleophilic addition of the ^–^SCF_3_ was in vain, the alternative approach involving “sulfur transfer reaction” and subsequent electrophilic trifluoromethylation with Togni reagent provided target products in high yield via a one-pot procedure. The structure of representative enantiomerically pure imidazol-2-yl trifluoromethyl sulfide was confirmed by X-ray analysis.

Introduction of fluoroalkyl substituent(s) into organic molecules remarkably tunes their physio-chemical and biological behavior.^1^ In this context, the SCF_3_ group characterized by the highest Hansch parameter (π = 1.44) has attracted special attention due to its extraordinary lipophilicity and highly electron-withdrawing character.^2^ Consequently, numerous methods have been developed for the synthesis of compounds functionalized with the trifluoromethylthiol unit,^3^ and various heterocyclic systems of significance as new agrochemicals, medicinal targets, and functional materials are known.^4^

Despite enormous interest in manifold *N*-heterocycles decorated with a trifluoromethylthiol group, the title 2-CF_3_S-imidazoles are known only to a limited extent (Scheme 1). In early work by Haber, highly toxic bis(trifluoromethyl) disulfide was applied as trifluoromethylthiolating agent in reactions with *N*-(tetrahydropyran-2-yl)-masked 4,5-diaryl-imidazoles, by using organyllithiums as a base.^5^ More recently, Gravatt and Ghosh disclosed elegant photoredox Ni-catalyzed trifluoromethylthiolation protocol employing (hetero)aryl iodides and AgSCF_3_, and the method was successfully applied for the reaction with 2-iodo-1-methylimidazole (R = Me).^6^ In a similar NCS-mediated approach, Chen and co-workers accessed 2-CF_3_S-1-phenylimidazole (R = Ph), which was further applied as an efficient agent for the dehydroxytrifluoromethylthiolation of alcohols.^7^ In addition, synthesis and phase-transfer catalytic properties of some 1,3-dialkylimidazolium salts bearing the CF_3_S group have also been documented.^8^ Finally, two syntheses of a model 1-methyl-2-CF_3_S-benzimidazole through (i) photocatalytic trifluoromethylation of 1-methyl-benzimidazole-2-thiol with CF_3_I and (ii) Cu-mediated trifluoromethylthiolation of the respective bromide are known.^9^ However, neither of the mentioned works reported on the synthesis of fully substituted imidazole derivatives nor exploited imidazole *N*-oxides as key building blocks.

**Scheme 1:**
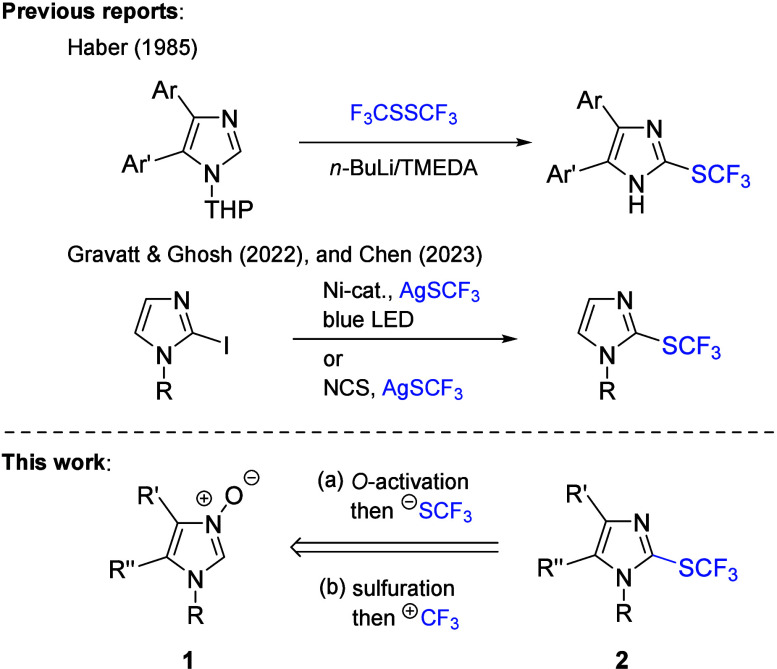
Reported Syntheses of 2-CF_3_S-Imidazoles and the Approaches Examined Herein

In continuation of our study aimed at the development of convenient synthetic methods toward fluoroalkylated azoles,^10^ we turned our attention to readily available aldonitrone-like imidazole *N*-oxides **1** recognized as superior building blocks for C(2)-functionalization of the imidazole ring.^11^ Thus, both nucleophilic trifluoromethylthiolation at the C(2) preceded by *O*-activation of the *N*-oxide function (route a) as well as an indirect pathway comprising the sulfuration/*S*-trifluoromethylation reaction sequence (route b) should be examined (Scheme 1). Here, we report a simple and efficient method for the preparation of fully substituted 2-CF_3_S-imidazoles of type **2** via the latter, two-step one-pot protocol.

For the initial experiments, 1-benzyl-4,5-dimethylimidazole *N*-oxide (**1a**), readily available by condensation of diacetyl monoxime and *N*-benzylformaldimine,^12^ was selected as the model starting material. Prompted by the seminal work of Kuninobu on direct trifluoromethylthiolation of some six-membered *N*-oxides with AgSCF_3_,^13^ imidazole derivative **1a** was first checked under the analogous reaction conditions, but no desired product **2a** could be detected in the crude mixture (Scheme 2a). Brief screening of the reaction conditions with respect to solvents (DCM, EtOAc, and DMF) and activating agents (TsCl, 2,4-dinitrobenzenesulfonyl chloride, and 1-adamantanecarbonyl chloride) revealed insufficient electrophilic character of the activated imidazole *N*-oxide to trap the SCF_3_ anion, and in all of the attempts, unreacted **1a** along with small amounts of the parent heterocycle, that is, 1-benzyl-4,5-dimethylimidazole, were identified in mother liquors.

**Scheme 2:**
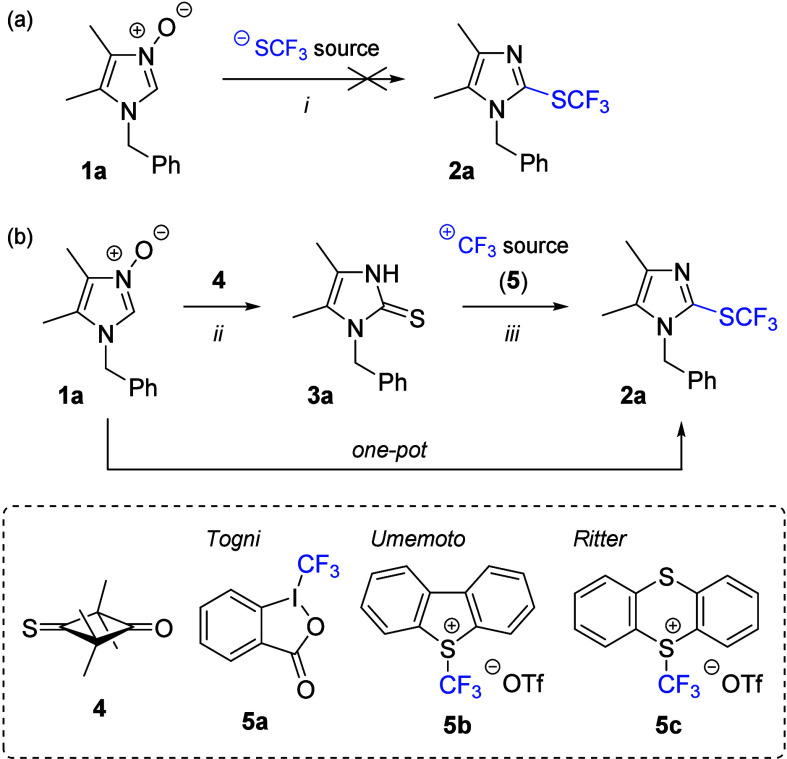
Initial Experiments with Imidazole *N*-Oxide **1a** Reagents and conditions: (i) 2,4-(NO_2_)_2_C_6_H_3_SO_2_Cl (1.1 equiv), AgSCF_3_ (1.1 equiv), MeCN, rt, overnight; (ii) thione **4** (1.1 equiv), DCM, rt, 2 h, 95% yield; and (iii) **5** (1.5 equiv); for solvent, temperature, and the additives used, see Table 1.

Next, we turned attention to an alternative two-step approach through electrophilic trifluoromethylation of enolizable imidazole-2-thione **3a** as a key intermediate (Scheme 2b). The requisite **3a** was prepared in 95% yield via a known sulfur-transfer method based on sequential (3 + 2)-cycloaddition/cycloreversion reactions of 2-unsubstituted imidazole *N*-oxide **1a** with strained thiocarbonyl agents such as 2,2,4,4-tetramethyl-3-thioxocyclobutanone (**4**).^12^ With a model imidazole-2-thione **3a** in hand, three selected reagents **5a**–**5c**, recommended for electrophilic trifluoromethylations of the thiol group,^14^ were checked as reaction partners under mild, neutral conditions (DCM, rt), to provide the target 2-CF_3_S-imidazole derivative **2a** in 36% and 25% yields by using Togni (**5a**) and Umemoto (**5b**) reagents, respectively (Table 1, entries 1–2; for the complete optimization table, see the Supporting Information). Taking into account the observed yields and the formation of a rather complex mixture in the latter case, the optimization was continued with Togni reagent.

**Table 1:** Trifluoromethylation of Imidazole-2-thione **3a**^a^

entry	reagent	additive	solvent	temp	yield
1	**5a**		DCM	rt	36
2	**5b**		DCM	rt	25
3	**5c**		DCM	rt	0
4	**5a**		MeCN	rt	28
5	**5a**	TsOH (cat.)	DCM	rt	40
6	**5a**	HCl (cat.)	DCM	rt	44
7	**5a**	HCl (excess)	MeOH	rt	67
8	**5a**	HCl (excess)	MeOH	0 °C	72
9	**5a**	HCl (excess)	MeOH	–30 °C	91
10	**5a**	HCl (excess)	MeOH	–30 °C	83^b^

a Reaction conditions: **3a** (0.5 mmol), **5** (0.75 mmol), solvent (5.0 mL), 15 min, inert atmosphere (Ar). Isolated yield of **2a**.

b One-pot reaction starting from *N*-oxide **1a** (0.5 mmol): thione **4** (0.55 mmol), DCM, rt, 2 h, then **5a** (0.75 mmol), MeOH/HCl, −30 °C, 15 min.

Further analysis of the reaction parameters and the role of additives indicated the beneficial presence of Brønsted acids (entries 5–7),^15^ at low temperatures (entries 8–9), and by using dry MeOH saturated with HCl as the reaction medium, at −30 °C, the final product **2a** was obtained in an excellent yield of 91% (entry 9). Furthermore, the one-pot protocol starting with *N*-oxide **1a** was also checked, and, in that case, the first formed crude imidazole-2-thione **3a** was subsequently applied for the optimized trifluoromethylation step to afford **2a** in high 83% overall yield (for two steps; entry 10) after standard chromatographic purification on neutral alumina.

With the optimized conditions in hand, a series of imidazole *N*-oxides **1a**–**1p** was examined to check the scope of the devised one-pot protocol (Scheme 3). In all of the cases, the expected products **2a**–**2p** were obtained in high yield (64–98%) irrespective of the nature of the substituents and functional groups present in the starting material. Thus, (cyclo)alkyl and aryl substituents located at either N(1) or C(4)/C(5) positions of the imidazole ring (**2a**–**2d**, **2h**–**2l**) as well as hydroxy (**2e**), ketone (**2m**–**2o**), ester (**2p**), and amide (**2f**) groups were tolerated, leading to desired products in a fully chemoselective manner. Notably, no epimerization was observed under the applied conditions in reaction with enantiomerically pure imidazole *N*-oxide **1g** bearing a stereogenic center at the benzylic position. The structure of final products was established based on suitable NMR analyses, supplemented with 2D measurements in certain cases. Particularly, the presence of two diagnostic quartets (^1^*J*_C–F_ ≈ 312 Hz and ^3^*J*_C–F_ ≈ 3.0 Hz) located at δ = 128.0–128.4 and 125.5–129.7, respectively, in the ^13^C NMR spectra of **2a**–**2p** confirmed the presence of the C(2)SCF_3_ unit in the obtained series. In addition, in ^19^F NMR spectra, singlet absorptions were found at the region typical for the trifluoromethylthiol group (δ ≈ −45.0). Finally, single-crystal X-ray analysis of chiral *N*-(2-hydroxy-1-phenylethyl)-functionalized analogue **2g** unambiguously confirmed its molecular structure (see the Supporting Information).

**Scheme 3:**
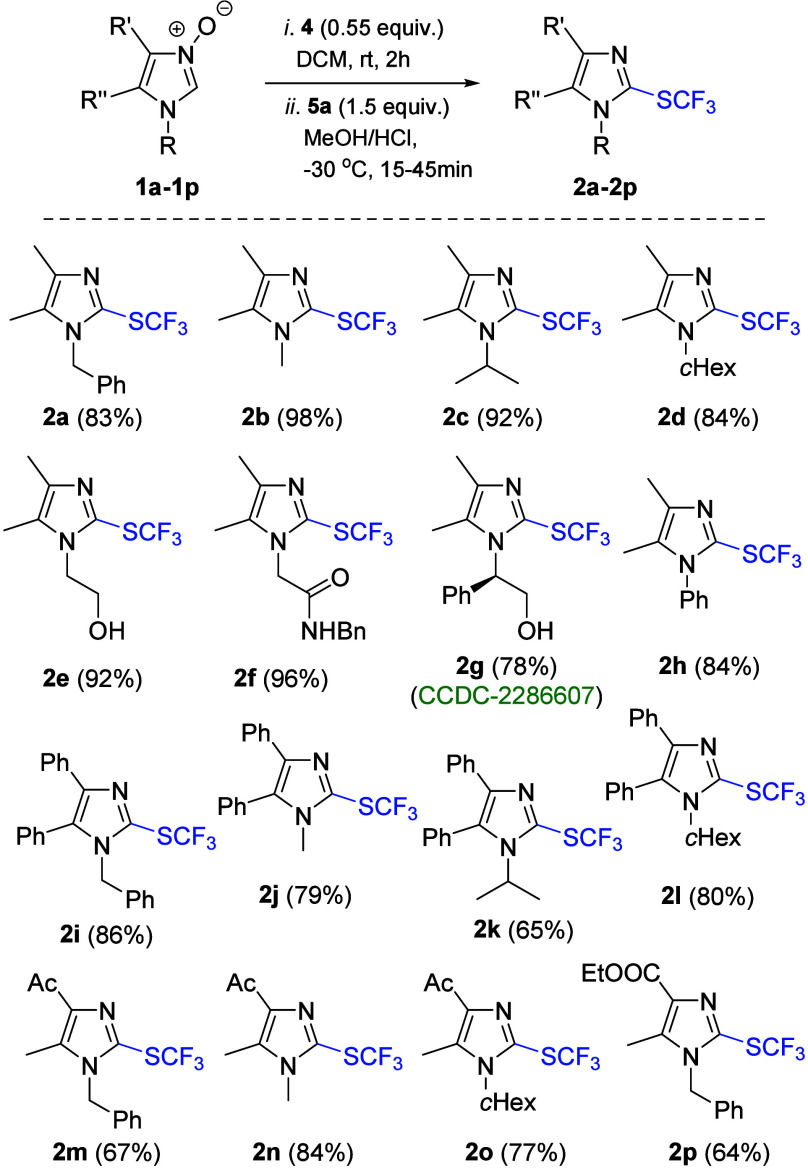
Scope of Imidazole *N*-Oxides **1** in the One-Pot Synthesis of Imidazol-2-yl Trifluoromethyl Sulfides **2**

In order to check whether the devised protocol can be applied for a larger scale, the synthesis of 2-CF_3_S-imidazole **2i** was repeated starting with 1.63 g (5.0 mmol) of *N*-oxide **1i** (Scheme 4). Gratifyingly, the desired sulfide was obtained in comparable yield (1.82 g, 89%), despite a smaller excess (1.2 equiv) of trifluoromethylating agent **5a** used in this experiment. Furthermore, bis(imidazole *N*-oxide) **1q** was involved in the study; by using excesses of *S*-transferring agent **4** (0.55 equiv/*N*-oxide group) and **5a** (2.0 equiv/HS-group), the expected bis-trifluoromethylthiolated product **2q** was isolated in fair 52% yield.

**Scheme 4:**
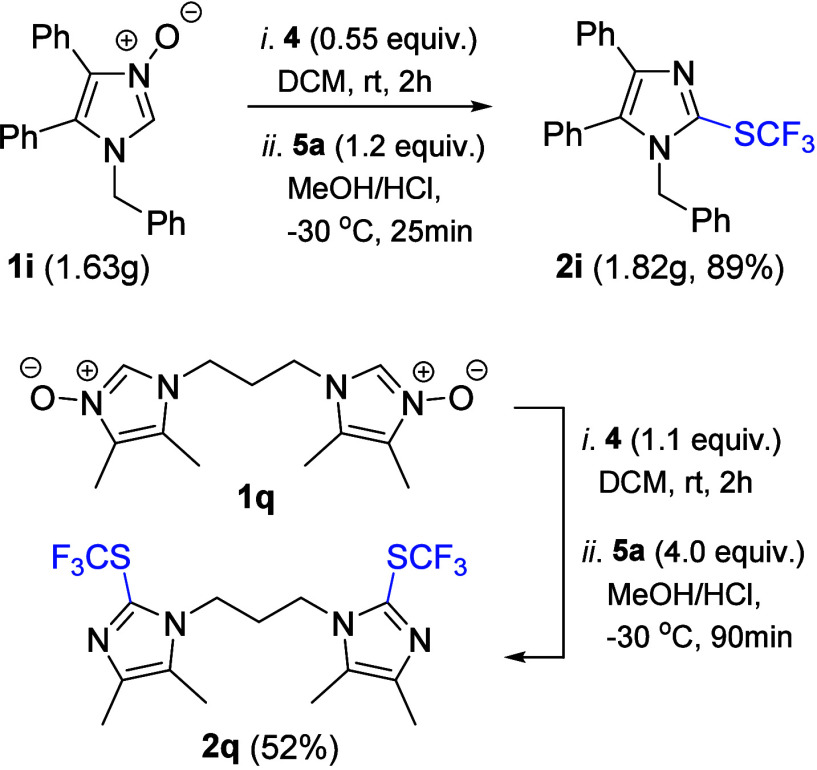
Gram-Scale Synthesis of **2i** and Preparation of Bis-sulfide **2q**

In summary, fully substituted 1,4,5-aryl/alkyl 2-trifluoromethylsulfanyl imidazoles in overall yields typically exceeding 80% through a two-step one-pot protocol are reported. The devised method based on sulfur-transfer followed by the electrophilic trifluoromethylation reaction sequence exploits readily available and cheap building blocks, that is, imidazole *N*-oxides, cyclobutanethione derivative, and Togni reagent, and it is characterized by excellent chemoselectivity as demonstrated in reactions with hydroxy-, ketone-, ester-, and amide-functionalized starting materials. The presented method can also be recommended for the synthesis of more complex imidazole systems, including chiral and bis-trifluoromethylthiolated analogues.

## Experimental Section

### General

All commercially available reagents and solvents were used as received. Products were purified by standard column chromatography (CC) on neutral alumina by using freshly distilled solvents. NMR spectra were taken at 600 MHz (^1^H) and 151 MHz (^13^C) and reported relative to the solvent residual peaks [CDCl_3_: ^1^H NMR, δ = 7.26; ^13^C NMR, δ = 77.16] or at 565 MHz (^19^F) and referenced to CFCl_3_ (δ = 0.00) used as the external standard. Structural assignments were made with additional information from gCOSY, gHSQC, and gHMBC experiments. IR spectra were taken with an Agilent Cary 630 FTIR spectrometer, in neat. ESI–MS was performed with a Varian 500-MS LC Ion Trap. Combustion analyses were obtained with a Vario EL III (Elementar Analysensysteme GmbH) instrument. Melting points were determined in capillaries with a MEL-TEMP apparatus (Laboratory Devices), and they are uncorrected.

#### General Procedure for the Synthesis of Sulfides **2**

To a solution of imidazole *N*-oxide **1** (0.5 mmol) in dry DCM (4.0 mL) was added dropwise a solution of 2,2,4,4-tetramethyl-3-thioxocyclobutanone (**4**, 86 mg, 0.55 mmol, 1.1 equiv) in DCM (3.0 mL), and the mixture was stirred at room temperature for 2 h. The solvent was removed in vacuo, the residue was dissolved in MeOH/HCl_(sat.)_ (2.0 mL), cooled to −30 °C, and a solution of **5a** (237 mg, 0.75 mmol, 1.5 equiv) in MeOH/HCl_(sat.)_ (3.0 mL) was added under an inert atmosphere. After the intermediate imidazole-2-thione **3** was fully consumed (TLC monitoring, typically 15–30 min), solvents were removed in vacuo, and the product **2** was purified by column chromatography (CC) on neutral alumina.

##### 1-Benzyl-4,5-dimethyl-2-[(trifluoromethyl)sulfanyl]-1*H*-imidazole (**2a**)

Reaction time 15 min; CC (alumina, DCM gradient DCM/EtOAc 4:1); colorless solid, 119 mg (83%); mp 52–54 °C. ^1^H NMR (600 MHz, CDCl_3_) δ: 7.33–7.27 (m, 3H), 6.93–6.92 (m, 2H), 5.30 (s, 2H), 2.23 (s, 3H), 2.04 (s, 3H). ^13^C{^1^H} NMR (151 MHz, CDCl_3_): δ 138.3, 136.0, 129.3, 129.1, 128.2 (q, ^1^*J*_C–F_ = 312.2 Hz), 128.0, 126.5 (q, ^3^*J*_C–F_ = 2.8 Hz), 126.1, 48.6, 13.1, 9.9. ^19^F NMR (565 MHz, CDCl_3_): δ −47.2 (s, CF_3_). IR (neat): ν 3030, 2959, 2929, 1572, 1494, 1453, 1401, 1148, 1092 cm^–1^. MS (ESI) *m*/*z*: 287.1 (100, [M + H]^+^). Anal. Calcd for C_13_H_13_F_3_N_2_S: C, 54.53; H, 4.58; N, 9.78; S, 11.20. Found: C, 54.48; H, 4.68; N, 9.60; S, 11.23.

## Data Availability

The data underlying this study are available in the published article and its Supporting Information.

## Supporting Information Available

Synthetic procedures, characterization data, and NMR spectra of all compounds (PDF)
FAIR data, including the primary NMR FID files, for compounds **2a**–**2q** (ZIP)
