# Supplementary material for: One-Pot Telescoping S-Transfer and Trifluoromethylation for the Synthesis of 2-CF3S-Imidazoles with N-Oxides as Convenient Precursors
Source: J Org Chem. 2024 Sep 30;89(20):15331–5. doi: 10.1021/acs.joc.4c01761 (PMC11494641; doi:10.1021/acs.joc.4c01761)
Supplement: Supplementary file 1 — jo4c01761_si_001.pdf [file jo4c01761_si_001.pdf]

Supporting Information

for

One-pot telescoping *S*-transfer and trifluoromethylation for the synthesis of 2-CF_3S-imidazoles with *N*-oxides as convenient precursors

Wiktor K. Poper,[a,b] Jun-An Ma,[c,*] Marcin Jasiński[a,*]

[a] *University of Lodz, Faculty of Chemistry, Department of Organic and Applied Chemistry, 91403 Łódź, Poland;*
[b] *The University of Lodz Doctoral School of Exact and Natural Sciences, Banacha 12/16, 90237 Łódź, Poland*
[c] *Department of Chemistry, Tianjin Key Laboratory of Molecular Optoelectronic Sciences, Frontiers Science Center for Synthetic Biology (Ministry of Education), and Tianjin Collaborative Innovation Centre of Chemical Science and Engineering, Tianjin University, Tianjin 300072, P. R. of China*

* Corresponding authors:

Jun-An Ma – *Department of Chemistry, Tianjin University, Tianjin 300072, P. R. of China;* https://orcid.org/0000-0002-3902-6799; Phone: 0086-22-27407286; Fax: 0086-22-27403475; E-mail: majun_an68@tju.edu.cn

Marcin Jasiński – *Faculty of Chemistry, University of Lodz, 91403 Łódź, Poland;* https://orcid.org/0000-0002-8789-9690; Phone: 48-42-6355766; Email: marcin.jasinski@chemia.uni.lodz.pl

Content

1. General information S2
2. Synthetic procedures and characterization data S3
3. Copies of NMR spectra S10
4. Crystallographic analysis S27
5. References S33

1. General information

Experimental procedures: Commercially available starting materials and solvents (DCM, MeOH) were used as received; if not stated otherwise, reactions were carried out under inert atmosphere of argon, in flame-dried flasks; subsequent manipulations were conducted in air. Products were purified by standard column chromatography (CC) on neutral alumina by using freshly distilled solvents as eluents or by recrystallization from appropriate solvents. Melting points were determined in capillaries with a MEL-TEMP II apparatus (Laboratory Devices), and are uncorrected. Optical rotations were determined with an Anton Paar MCP 500 polarimeter at the temperatures indicated. NMR spectra were measured on a Bruker Avance III or Bruker AvanceNeo instruments (^{1}H at 600 MHz, ^{13}C at 151 MHz, and ^{19}F at 565 MHz); chemical shifts are reported relative to solvent residual peaks [for $CDCl_3$: ^{1}H NMR: δ = 7.26, ^{13}C NMR: δ = 77.16; for DMSO-d_6: ^{1}H NMR: δ = 2.50, ^{13}C NMR: δ = 39.52] or to $CFCl_3$ (^{19}F NMR: δ = 0.00) used as external standard. Assignments and multiplicity of the signals in ^{13}C NMR spectra were deduced based on 2D measurements (HMQC). The IR spectra were taken with an Agilent Cary 630 FTIR spectrometer, in neat. ESI-MS were performed with a Varian 500-MS LC Ion Trap; high resolution MS (ESI-TOF) measurements were performed with a Synapt G2-Si mass spectrometer (Waters). Combustion analyses were obtained with a Vario EL III (Elementar Analysensysteme GmbH) instrument.

Starting materials: The starting imidazole *N*-oxides **1a**-**1q** were prepared following the literature protocols, through condensation of the corresponding formaldimines with α-hydroxyiminoketones, either using EtOH or glacial acetic acid as a solvent.[1] 2,2,4,4-Tetramethyl-3-thioxocyclobutanone (**4**) was prepared by thionation of the respective ketone with phosphorus pentasulfide.[2]

2. Synthetic procedures and characterization data

Synthesis of 1-benzyl-4,5-dimethylimidazole-2(3*H*)-thione (3a):[1a] To a solution of imidazole *N*-oxide **1a** (10.0 mmol, 2.00 g) in dry DCM (40 mL) was added dropwise a solution of 2,2,4,4-tetramethyl-3-thioxocyclobutanone (**4**, 11.0 mmol, 1.72 g, 1.1 equiv.) in DCM (20 mL) and the mixture was stirred at room temperature for 2h. After solvent was removed in vacuo, the resulting material was washed with hexane (4 x 50 mL) and the product was filtered off to give spectroscopically pure imidazole-2-thione **3a** (2.07 g, 95%) as a colorless solid. Mp 225-227 °C (Ref[1a]: Mp 227-228 °C). ^{1}H NMR (600 MHz, $CDCl_3$) δ 10.81 (s_{br}, 1H), 7.33-7.30 (m, 2H), 7.28-7.24 (m, 3H), 5.29 (s, 2H), 2.07 (s, 3H), 1.91 (s, 3H).

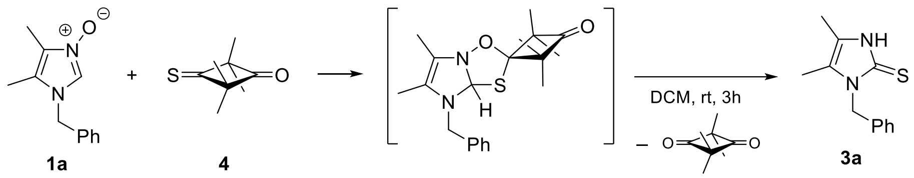

Scheme S1. Synthesis of model imidazole-2(3*H*)-thione **3a** from the corresponding *N*-oxide **1a** via '*sulfur transfer reaction*' using 2,2,4,4-tetramethyl-3-thioxocyclobutanone (**4**).

Synthesis of model sulfide 2a – optimization procedure: In round-bottomed flask equipped with a stirring bar a model imidazole-2-thione **3a** (0.5 mmol) was placed and the solvent (2.0 mL) was added. The flask was sealed with a septum under an atmosphere of argon, and cooled or heated, if necessary (see Table S1). Then additive and a solution of trifluoromethylating agent **5** (0.75 mmol, 1.5 equiv.) in a corresponding solvent (3.0 mL) were added to the reaction mixture, and the resulting was stirred for required time. After solvents were removed under reduced pressure the product **2a** was purified by column chromatography on neutral alumina.

Table S1. Synthesis of model trifluoromethyl-sulfide **2a**.

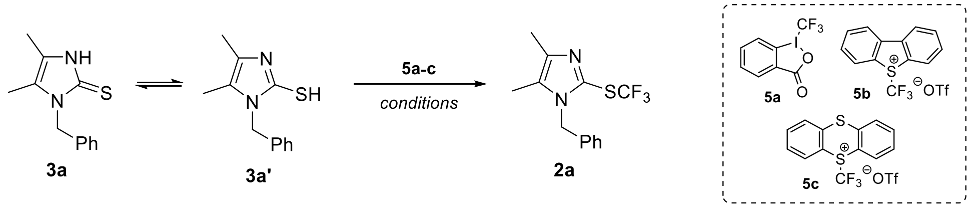

Entry	CF_3-reagent	additive	solvent	temp [°C]	time [min]	yield [%]
1	**5a**	-	DCM	rt	15	36
2	**5a**	-	DCM	rt	120	38
3	**5b**	-	DCM	rt	15	25
4	**5b**	-	DCM	rt	120	24
5	**5c**	-	DCM	rt	15	0
6	**5c**	-	DCM	rt	120	0
7	**5c**	Et_3N	DMF	40	15	0

8	**5a**	-	MeCN	rt	15	28
9	**5a**	TsOH (cat.)	DCM	rt	15	40
10	**5a**	TsOH (cat.)	DCM	0	15	44
11	**5a**	TsOH (cat.)	DCM	–30	15	48
12	**5a**	HCl (cat.)	DCM	rt	15	44
13	**5a**	HCl (cat.)	DCM	–30	15	52
14	**5a**	TsOH (cat.)	MeOH	–30	15	50
15	**5a**	HCl (excess)	MeOH	rt	15	67
16	**5a**	HCl (excess)	MeOH	0	15	72
17	**5a**	HCl (excess)	MeOH	–30	15	91
18	**5a**	HCl (excess)	MeOH	–40	15	90
16	**5a** (*one-pot* starting from **1a**) *	HCl (excess)	MeOH	–30	15	83

* One-pot reaction starting from *N*-oxide **1a** (0.5 mmol): thione **4** (0.55 mmol), DCM, rt, 2h, then **5a** (0.75 mmol), MeOH/HCl, –30 °C, 15 min.

General one-pot procedure for synthesis of 2-CF_3S-imidazole derivatives 2a-2q: To a solution of imidazole *N*-oxide **1** (0.5 mmol) in dry DCM (4.0 mL) was added dropwise a solution of 2,2,4,4-tetramethyl-3-thioxocyclobutanone (**4**, 86 mg, 0.55 mmol, 1.1 equiv.) in DCM (3.0 mL) and the mixture was stirred at room temperature for 2h. The solvent was removed in vacuo, the residue was dissolved in $MeOH/HCl_{(sat.)}$ (2.0 mL), cooled to –30 °C, and a solution of **5a** (237 mg, 0.75 mmol, 1.5 equiv.) in $MeOH/HCl_{(sat.)}$ (3.0 mL) was added under inert atmosphere. After the intermediate imidazole-2-thione **3** was fully consumed (TLC monitoring, typically 15-30 min), solvents were removed in vacuo, and the product **2** was purified by a column chromatography (CC) on neutral alumina.

1-Benzyl-4,5-dimethyl-2-[(trifluoromethyl)sulfanyl]-1*H*-imidazole (**2a**):

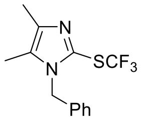

2a

Reaction time 15 min; CC (alumina, DCM gradient DCM/EtOAc 4:1); colorless solid, 119 mg (83%); mp 52-54 °C. ^{1}H NMR (600 MHz, $CDCl_3$) δ 7.33-7.27 (m, 3H), 6.93-6.92 (m, 2H), 5.30 (s, 2H), 2.23 (s, 3H), 2.04 (s, 3H). ^{13}C{1H} NMR (151 MHz, $CDCl_3$) δ 138.3, 136.0, 129.3, 129.1, 128.2 (q, $^1J_{C-F}$ = 312.2 Hz), 128.0, 126.5 (q, $^3J_{C-F}$ = 2.8 Hz), 126.1, 48.6, 13.1, 9.9. ^{19}F NMR (565 MHz, $CDCl_3$): δ –42.5 (s, CF_3). IR (neat) ν 2930, 1573, 1495, 1454, 1402, 1357, 1148, 1092 cm^{-1}. MS (ESI) *m/z*: 287.1 (100, $[M+H]^+$). Anal. Calcd for $C_{13}H_{13}F_3N_2S$: C, 54.53; H, 4.58; N, 9.78; S 11.20. Found: C, 54.48; H, 4.68; N, 9.60; S, 11.23.

1,4,5-Trimethyl-2-[(trifluoromethyl)sulfanyl]-1*H*-imidazole (**2b**):

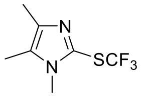

2b

Reaction time 15 min; CC (alumina, DCM gradient DCM/EtOAc 4:1); colorless solid, 103 mg (98%); mp 76-78 °C. ^{1}H NMR (600 MHz, $CDCl_3$) δ 3.61 (s, 3H), 2.17 (s, 6H). ^{13}C{1H} NMR (151

MHz, $CDCl_3$) δ 137.5, 129.4, 128.3 (q, $^1J_{C-F}$ = 311.9 Hz), 125.8 (q, $^3J_{C-F}$ = 3.3 Hz), 31.9, 12.9, 9.7. ^{19}F NMR (565 MHz, $CDCl_3$): δ –42.7 (s, CF_3). IR (neat) ν 1569, 1439, 1394, 1111 cm^{-1}. MS (ESI) *m/z*: 211.2 (100, $[M+H]^+$). Anal. Calcd for $C_7H_9F_3N_2S$: C, 40.00; H, 4.32; N, 13.33; S, 15.25. Found: C, 40.04; H, 4.38; N, 13.41; S, 15.41.

1-Isopropyl-4,5-dimethyl-2-[(trifluoromethyl)sulfanyl]-1*H*-imidazole (**2c**):

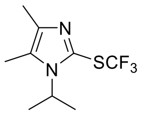

2c

Reaction time 15 min; CC (alumina, DCM gradient DCM/EtOAc 4:1); colorless solid, 109 mg (92%); mp 32-34 °C. ^{1}H NMR (600 MHz, $CDCl_3$) δ 5.02 (sept, *J* = 7.0 Hz, 1H), 2.30 (s, 3H), 2.18 (s, 3H), 1.49 (d, *J* = 7.0 Hz, 6H). ^{13}C{1H} NMR (151 MHz, $CDCl_3$) δ 138.9, 128.17, 128.15 (q, $^1J_{C-F}$ = 311.9 Hz), 125.5 (q, $^3J_{C-F}$ = 3.3 Hz), 49.6, 21.7, 12.9, 11.0. ^{19}F NMR (565 MHz, $CDCl_3$): δ –41.6 (s, CF_3). IR (neat) ν 1502, 1446, 1331, 1170, 1100, 1025 cm^{-1}. HRMS (ESI-TOF) *m/z*: $[M+H]^+$ Calcd for $C_9H_{14}F_3N_2S$ 239.0830; Found 239.0827.

1-Cyclohexyl-4,5-dimethyl-2-[(trifluoromethyl)sulfanyl]-1*H*-imidazole (**2d**):

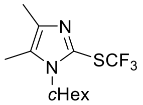

2d

Reaction time 15 min; CC (alumina, DCM gradient DCM/EtOAc 4:1); colorless solid, 117 mg (84%); mp 56-58 °C. ^{1}H NMR (600 MHz, $CDCl_3$) δ 4.53 (m_c, 1H), 2.30 (s, 3H), 2.17 (s, 3H), 1.98-1.90 (m, 4H), 1.84-1.80 (m, 1H), 1.78-1.74 (m, 1H), 1.46-1.38 (m, 2H), 1.26-1.18 (m, 1H). ^{13}C{1H} NMR (151 MHz, $CDCl_3$) δ 138.7, 128.3, 128.2 (q, $^1J_{C-F}$ = 311.7 Hz), 125.8 (br, C-2), 58.4, 31.9, 26.3, 25.4, 12.9, 11.3. ^{19}F NMR (565 MHz, $CDCl_3$): δ –43.0 (s, CF_3). IR (neat) ν 1566, 1402, 1345, 1323, 1137, 1096 cm^{-1}. MS (ESI) *m/z*: 279.3 (100, $[M+H]^+$). Anal. Calcd for $C_{12}H_{17}F_3N_2S$: C, 51.78; H, 6.16; N, 10.06; S, 11.52. Found: C, 51.74; H, 6.27; N, 10.01; S, 11.73.

1-(2-Hydroxyethyl)-4,5-dimethyl-2-[(trifluoromethyl)sulfanyl]-1*H*-imidazole (**2e**):

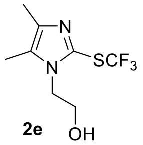

2e

Reaction time 30 min; CC (alumina, DCM/EtOAc 4:1 gradient 1:1); colorless solid, 110 mg (92%); mp 104-106 °C. ^{1}H NMR (600 MHz, DMSO-d_6) δ 4.97 (t, *J* = 5.4 Hz, 1H, OH), 4.10 (t, *J* = 5.8 Hz, 2H), 3.56 (pseudo-q, *J* ≈ 5.6 Hz, 2H), 2.19 (s, 3H), 2.09 (s, 3H). ^{13}C{1H} NMR (151 MHz, DMSO-d_6) δ 136.3, 129.3, 128.2 (q, $^1J_{C-F}$ = 311.8 Hz), 124.6 (br, C-2), 59.9, 46.8, 12.6, 9.2. ^{19}F NMR (565 MHz, DMSO-d_6): δ –42.3 (s, CF_3). IR (neat) ν 3179, 1588, 1413, 1345, 1111, 1073 cm^{-1}. MS (ESI) *m/z*: 241.2 (100, $[M+H]^+$). Anal. Calcd for $C_8H_{11}F_3N_2OS$: C, 40.00; H, 4.62; N, 11.66; S, 13.34. Found: C, 39.95; H, 4.58; N, 11.55; S, 13.14.

N-benzyl-2-(4,5-dimethyl-2-[(trifluoromethyl)sulfanyl]-1*H*-imidazol-1-yl)acetamide (**2f**):

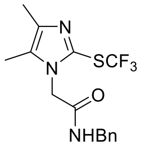

2f

Reaction time 15 min; CC (alumina, DCM gradient DCM/EtOAc 1:1); colorless solid, 165 mg (96%); mp 140-141 °C. ^{1}H NMR (600 MHz, $CDCl_3$) δ 7.33-7.28 (m, 3H), 7.21-7.26 (m, 2H), 6.03 (t_{br}, $J \approx 5.9$ Hz, 1H, NH), 4.76 (s, 2H), 4.44 (d, $J = 5.9$ Hz, 2H), 2.14 (s, 3H), 2.13 (s, 3H). ^{13}C{1H} NMR (151 MHz, $CDCl_3$) δ 165.9, 138.7, 137.5, 129.5, 129.0, 127.99, 127.96 (q, $^{1}J_{C\text{-}F} = 312.7$ Hz), 127.9, 126.8 (q, $^{3}J_{C\text{-}F} = 2.9$ Hz), 48.4, 43.9, 12.9, 9.7. ^{19}F NMR (565 MHz, $CDCl_3$): δ –42.2 (s, CF_3). IR (neat) ν 3273, 1659, 1558, 1402, 1297, 1252, 1163, 1141, 1103 cm^{-1}. MS (ESI) *m/z*: 344.2 (100, $[M+H]^+$). Anal. Calcd for $C_{15}H_{16}F_3N_3OS$: C, 52.47; H, 4.70; N, 12.24; S, 9.34. Found: C, 52.50; H, 4.73; N, 12.00; S, 9.34.

(*R*)-2-{4,5-dimethyl-2-[(trifluoromethyl)sulfanyl]-1*H*-imidazol-1-yl}-2-phenylethanol (**2g**):

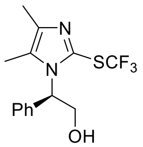

2g

Reaction time 15 min; CC (alumina, DCM gradient DCM/EtOAc 1:1); colorless solid, 123 mg (78%); mp 140-141 °C. $[\alpha]^{20}_{D} = -39.4°$ (c = 0.185, MeOH). ^{1}H NMR (600 MHz, $CDCl_3$) δ 7.37-7.30 (m, 3H), 7.16-7.14 (m, 2H), 6.07 (dd, $J = 5.2, 8.2$ Hz, 1H), 4.56 (dd, $J = 5.2, 11.6$ Hz, 1H), 4.30 (dd, $J = 8.2, 11.6$ Hz, 1H), 3.54 (s_{br}, 1H), 2.08 (s, 3H), 1.80 (s, 3H). ^{13}C{1H} NMR (151 MHz, $CDCl_3$) δ 138.5, 136.3, 129.0, 128.7, 128.27 (br, C-2), 128.25, 128.1 (q, $^{1}J_{C\text{-}F} = 312.3$ Hz), 126.7, 62.8, 61.4, 12.9, 11.0. ^{19}F NMR (565 MHz, $CDCl_3$): δ –42.3 (s, CF_3). IR (neat) ν 1502, 1446, 1413, 1371, 1331, 1156, 1100, 1047 cm^{-1}. HRMS (ESI-TOF) *m/z*: $[M+H]^+$ Calcd for $C_{14}H_{16}F_3N_2OS$ 317.0935; Found 317.0938.

Crystals of **2g** suitable for X-ray measurements were obtained from dichloromethane/hexane (1:1) mixture by slow evaporation of the solvent.

4,5-Dimethyl-1-phenyl-2-[(trifluoromethyl)sulfanyl]-1*H*-imidazole (**2h**):

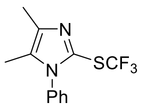

2h

Reaction time 30 min; CC (alumina, DCM gradient DCM/EtOAc 4:1); colorless solid, 114 mg (84%); mp 64-67 °C. ^{1}H NMR (600 MHz, $CDCl_3$) δ 7.52-7.51 (m, 3H), 7.17-7.16 (m, 2H), 2.28 (s, 3H), 2.00 (s, 3H). ^{13}C{1H} NMR (151 MHz, $CDCl_3$) δ 137.6, 136.2, 130.5, 129.51, 129.47, 128.2, 128.1 (q, $^{1}J_{C\text{-}F} = 312.0$ Hz), 126.9 (q, $^{3}J_{C\text{-}F} = 3.4$ Hz), 13.1, 10.1. ^{19}F NMR (565 MHz, $CDCl_3$): δ –42.3 (s, CF_3). IR (neat) ν 1595, 1498, 1413, 1163, 1103 cm^{-1}. MS (ESI) *m/z*: 273.2 (100, $[M+H]^+$). Anal. Calcd for $C_{12}H_{11}F_3N_2S$: C, 52.93; H, 4.07; N, 10.29; S, 11.77. Found: C, 53.03; H, 4.08; N, 10.12; S, 11.64.

1-Benzyl-4,5-diphenyl-2-[(trifluoromethyl)sulfanyl]-1*H*-imidazole (**2i**):

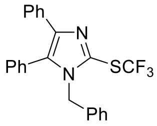

2i

Reaction time 30 min; CC (alumina, hexanes/DCM 1:4 gradient DCM); colorless solid, 176 mg (86%); mp 128-130 °C. ^{1}H NMR (600 MHz, $CDCl_3$) δ 7.51-7.49 (m, 2H), 7.43-7.40 (m, 1H), 7.37-7.34 (m, 2H), 7.26-7.16 (m, 8H), 6.82-6.79 (m, 2H), 5.25 (s, 2H). ^{13}C{1H} NMR (151 MHz, $CDCl_3$) δ 141.6, 136.4, 134.1, 133.5, 130.8, 130.3, 129.7 (q, $^3J_{C-F}$ = 2.8 Hz), 129.5, 129.2, 128.9, 128.33, 128.29 (q, $^1J_{C-F}$ = 312.1 Hz), 127.9, 127.3, 127.1, 126.3, 48.9. ^{19}F NMR (565 MHz, $CDCl_3$): δ –41.5 (s, CF_3). IR (neat) ν 1599, 1446, 1402, 1353, 1327, 1133, 1100 cm^{-1}. HRMS (ESI-TOF) *m/z*: [M+H]$^+$ Calcd for $C_{23}H_{18}F_3N_2S$ 411.1143; Found 411.1142.

1-Methyl-4,5-diphenyl-2-[(trifluoromethyl)sulfanyl]-1*H*-imidazole (**2j**):

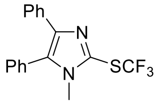

2j

Reaction time 30 min; CC (alumina, DCM); colorless solid, 132 mg (79%); mp 127-128 °C. ^{1}H NMR (600 MHz, $CDCl_3$) δ 7.50-7.47 (m, 5H), 7.35-7.34 (m, 2H), 7.22-7.16 (m, 3H), 3.61 (s, 3H). ^{13}C{1H} NMR (151,5 MHz, $CDCl_3$) δ 141.2, 134.3, 133.6, 130.6, 130.4, 129.5, 129.4, 129.3 (q, $^3J_{C-F}$ = 3.1 Hz), 128.4 (q, $^1J_{C-F}$ = 312.1 Hz), 128.3, 127.2, 127.1, 33.0. ^{19}F NMR (565 MHz, $CDCl_3$): δ –41.7 (s, CF_3). IR (neat) ν 1502, 1443, 1371, 1166, 1141, 1088 cm^{-1}. MS (ESI) *m/z*: 335.2 (100, [M+H]$^+$). Anal. Calcd for $C_{17}H_{13}F_3N_2S$: C, 61.07; H, 3.92; N, 8.38; S, 9.59. Found: C, 60.95; H, 4.00; N, 8.32; S, 9.65.

1-Isopropyl-4,5-diphenyl-2-[(trifluoromethyl)sulfanyl]-1*H*-imidazole (**2k**):

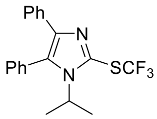

2k

Reaction time 30 min; CC (alumina, DCM); colorless solid, 117 mg (65%); mp 94-96 °C. ^{1}H NMR (600 MHz, $CDCl_3$) δ 7.52-7.46 (m, 3H), 7.39-7.36 (m, 4H), 7.18-7.12 (m, 3H), 4.77 (sept, *J* = 7.1 Hz, 1H), 1.41 (d, *J* = 7.1 Hz, 6H). ^{13}C{1H} NMR (151 MHz, $CDCl_3$) δ 141.7, 133.7, 133.3, 131.8, 131.2, 129.6, 129.1, 128.3 (q, $^1J_{C-F}$ = 312.1 Hz), 128.2, 128.1 (br, C-2), 126.98, 126.97, 50.6, 22.9. ^{19}F NMR (565 MHz, $CDCl_3$): δ –41.6 (s, CF_3). IR (neat) ν 1502, 1443, 1327, 1163, 1133, 1096 cm^{-1}. MS (ESI) *m/z*: 363.2 (100, [M+H]$^+$). Anal. Calcd for $C_{19}H_{17}F_3N_2S$: C, 62.97; H, 4.73; N, 7.73; S, 8.85. Found: C, 62.86; H, 4.67; N, 7.59; S, 8.73.

1-Cyclohexyl-4,5-diphenyl-2-[(trifluoromethyl)sulfanyl]-1*H*-imidazole (**2l**):

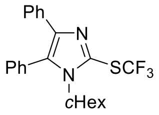

2l

Reaction time 30 min; CC (alumina, hexanes/DCM 1:4 gradient DCM); colorless solid, 161 mg (80%); mp 161-163 °C. ^{1}H NMR (600 MHz, $CDCl_3$) δ 7.52-7.46 (m, 3H), 7.39-7.34 (m, 4H), 7.17-7.11 (m, 3H), 4.30 (s_{br}, 1H), 1.85-1.76 (m, 6H), 1.62-1.58 (m, 1H), 1.26-1.21 (m, 2H), 1.03-0.93 (m, 1H). ^{13}C{1H} NMR (151 MHz, $CDCl_3$) δ 141.5, 133.7, 133.4, 131.7 (br)*, 131.2 (br, C-2), 129.6, 129.0, 128.3 (q, $^1J_{C\text{-}F}$ = 312.3 Hz), 128.2, 126.95, 126.93, 59.3, 33.1, 26.4, 25.2; *overlapped signals. ^{19}F NMR (565 MHz, $CDCl_3$): δ –41.7 (s, CF_3). IR (neat) ν 1443, 1327, 1133, 1100 cm^{-1}. MS (ESI) *m/z*: 403.3 (100, $[M+H]^+$). Anal. Calcd for $C_{22}H_{21}F_3N_2S$: C, 65.65; H, 5.26; N, 6.96; S, 7.97. Found: C, 65.70; H, 5.23; N, 6.82; S, 7.79.

4-Acetyl-1-benzyl-5-methyl-2-[(trifluoromethyl)sulfanyl]-1*H*-imidazole (**2m**):

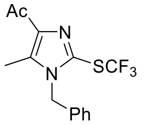

2m

Reaction time 1 h; CC (alumina, hexanes/DCM 1:4 gradient DCM); colorless solid, 105 mg (67%); mp 71-72 °C. ^{1}H NMR (600 MHz, $CDCl_3$) δ 7.36-7.29 (m, 3H), 6.94-6.93 (m, 2H), 5.36 (s, 2H), 2.62 (s, 3H), 2.49 (s, 3H). ^{13}C{1H} NMR (151 MHz, $CDCl_3$) δ 195.8, 140.2, 139.0, 134.7, 129.3, 129.0 (q, $^3J_{C\text{-}F}$ = 3.1 Hz), 128.5, 128.0 (q, $^1J_{C\text{-}F}$ = 312.2 Hz), 126.1, 48.4, 27.6, 11.4. ^{19}F NMR (565 MHz, $CDCl_3$): δ –41.5 (s, CF_3). IR (neat) ν 1670, 1543, 1454, 1432, 1398, 1353, 1141, 1096 cm^{-1}. MS (ESI) *m/z*: 315.2 (100, $[M+H]^+$). Anal. Calcd for $C_{14}H_{13}F_3N_2OS$: C, 53.50; H, 4.17; N, 8.91; S, 10.20. Found: C, 53.59; H, 4.29; N, 8.72; S, 10.19.

4-Acetyl-1,5-dimethyl-2-[(trifluoromethyl)sulfanyl]-1*H*-imidazole (**2n**):

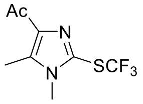

2n

Reaction time 1 h; CC (alumina, DCM); colorless solid, 99 mg (84%); mp 67-69 °C. ^{1}H NMR (600 MHz, $CDCl_3$) δ 3.70 (s, 3H), 2.61 (s, 3H), 2.59 (s, 3H). ^{13}C{1H} NMR (151 MHz, $CDCl_3$) δ 195.7, 139.7, 139.1, 128.6 (q, $^3J_{C\text{-}F}$ = 3.1 Hz), 128.2 (q, $^1J_{C\text{-}F}$ = 311.9 Hz), 31.8, 27.5, 11.3. ^{19}F NMR (565 MHz, $CDCl_3$): δ –41.6 (s, CF_3). IR (neat) ν 1670, 1539, 1372, 1141, 1088 cm^{-1}. HRMS (ESI-TOF) *m/z*: $[M+H]^+$ Calcd for $C_8H_{10}F_3N_2OS$ 239.0466; Found 239.0466.

4-Acetyl-1-cyclohexyl-5-methyl-2-[(trifluoromethyl)sulfanyl]-1*H*-imidazole (**2o**):

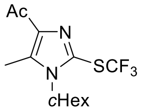

2o

Reaction time 2 h; CC (alumina, DCM); colorless solid, 118 mg (77%); mp 77-78 °C. ^{1}H NMR (600 MHz, $CDCl_3$) δ 4.58 (s_{br}, 1H), 2.73 (s, 3H), 2.57 (s, 3H), 2.08-2.01 (m, 2H), 1.97-1.93 (m, 2H), 1.86-1.84 (m, 2H), 1.80-1.77 (m, 1H), 1.47-1.40 (m, 2H), 1.29-1.22 (m, 1H). ^{13}C{1H} NMR (151 MHz,

$CDCl_3$) δ 196.0, 140.2, 138.2, 128.4 (br, C-2), 128.0 (q, $^1J_{C\text{-}F}$ = 312.1 Hz), 58.9, 31.5, 27.7, 26.3, 25.2, 12.4. ^{19}F NMR (565 MHz, $CDCl_3$): δ –41.9 (s, CF_3). IR (neat) ν 1674, 1536, 1431, 1364, 1141, 1095 cm^{-1}. MS (ESI) *m/z*: 329.2 (100, $[M+Na]^+$). Anal. Calcd for $C_{13}H_{17}F_3N_2OS$: C, 50.97; H, 5.59; N, 9.14; S, 10.47. Found: C, 51.17; H, 5.70; N, 8.93; S, 10.50.

1-Benzyl-4-ethoxycarbonyl-5-methyl-2-[(trifluoromethyl)sulfanyl]-1*H*-imidazole (**2p**):

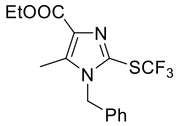

2p

Reaction time 90 min; CC (alumina, DCM gradient DCM/EtOAc 4:1); thick colorless oil, 110 mg (64%). ^{1}H NMR (600 MHz, $CDCl_3$) δ 7.35-7.29 (m, 3H), 6.93-6.92 (m, 2H), 5.37 (s, 2H), 4.40 (q, *J* = 7.1 Hz, 2H), 2.48 (s, 3H), 1.40 (t, *J* = 7.1 Hz, 3H). ^{13}C{1H} NMR (151 MHz, $CDCl_3$) δ 163.0, 140.6, 134.7, 132.7, 130.2 (q, $^3J_{C\text{-}F}$ = 3.3 Hz), 129.3, 128.4, 128.0 (q, $^1J_{C\text{-}F}$ = 312.5 Hz), 126.0, 60.9, 48.7, 14.6, 11.3. ^{19}F NMR (565 MHz, $CDCl_3$): δ –41.2 (s, CF_3). IR (neat) ν 1707, 1558, 1454, 1431, 1323, 1201, 1140, 1096 cm^{-1}. HRMS (ESI-TOF) *m/z*: $[M+H]^+$ Calcd for $C_{15}H_{16}F_3N_2O_2S$ 344.0885; Found 345.0891.

1,3-bis{4,5-dimethyl-2-[(trifluoromethyl)sulfanyl]-1*H*-imidazol-1-yl}propane (**2q**):

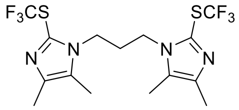

2q

According to the general protocol imidazole *N*-oxide **1q** (0.5 mmol) was treated with cyclobutanethione **4** (1.1 mmol), followed by Togni reagent (**5a**, 1.5 mmol); reaction time 2 h; CC (alumina, hexanes/DCM 1:4 gradient DCM); colorless solid, 112 mg (52%); mp 104-106 °C. ^{1}H NMR (600 MHz, $CDCl_3$) δ 4.07 (t, *J* = 7.8 Hz, 4H), 2.19 (s, 3H), 2.17 (s, 3H), 2.03 (m, 2H). ^{13}C{1H} NMR (151 MHz, $CDCl_3$) δ 138.6, 128.4, 128.0 (q, $^1J_{C\text{-}F}$ = 312.3 Hz), 125.5 (q, $^3J_{C\text{-}F}$ = 3.1 Hz), 42.1, 31.4, 13.0, 9.7. ^{19}F NMR (565 MHz, $CDCl_3$): δ –42.3 (s, $2CF_3$). IR (neat) ν 1577, 1402, 1364, 1156, 1096 cm^{-1}. HRMS (ESI-TOF) *m/z*: $[M+H]^+$ Calcd for $C_{15}H_{19}F_6N_4S_2$ 433.0955; Found 433.0962.

Gram-scale synthesis of 2-CF_3S-imidazole 2i: To a solution of imidazole *N*-oxide **1i** (1.63 g, 5.0 mmol) in dry DCM (40 mL) was added dropwise a solution of 2,2,4,4-tetramethyl-3-thioxocyclobutanone (**4**, 5.2 mmol, 0.80 g, 1.04 equiv.) in DCM (30 mL) and the mixture was stirred at room temperature for 2h. The solvent was removed in vacuo, the residue was dissolved in MeOH/$HCl_{(sat.)}$ (20 mL), cooled to –30 °C, and a solution of **5a** (6.0 mmol, 1.90 g, 1.2 equiv.) in MeOH/$HCl_{(sat.)}$ (30 mL) was added under inert atmosphere. After the intermediate imidazole-2-thione **3i** was fully consumed (TLC monitoring, 40 min), solvents were removed in vacuo, and the product was purified by a column chromatography (hexanes/DCM 1:4 gradient DCM) on neutral alumina to give **2i** (1.82 g, 89%) as a colorless solid.

3. Copies of NMR spectra

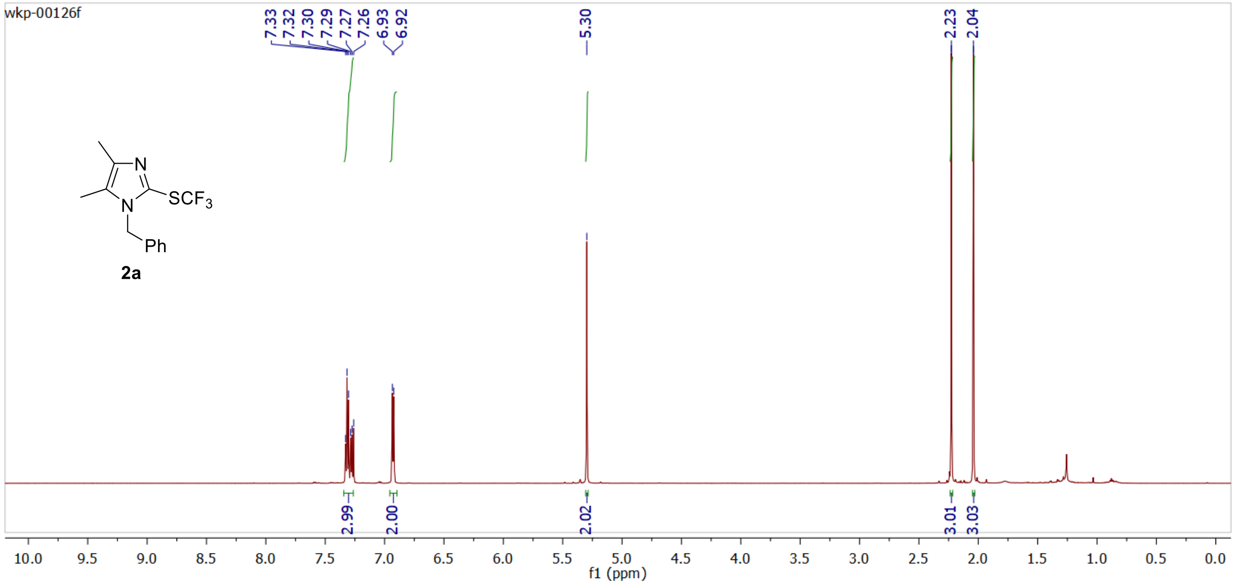

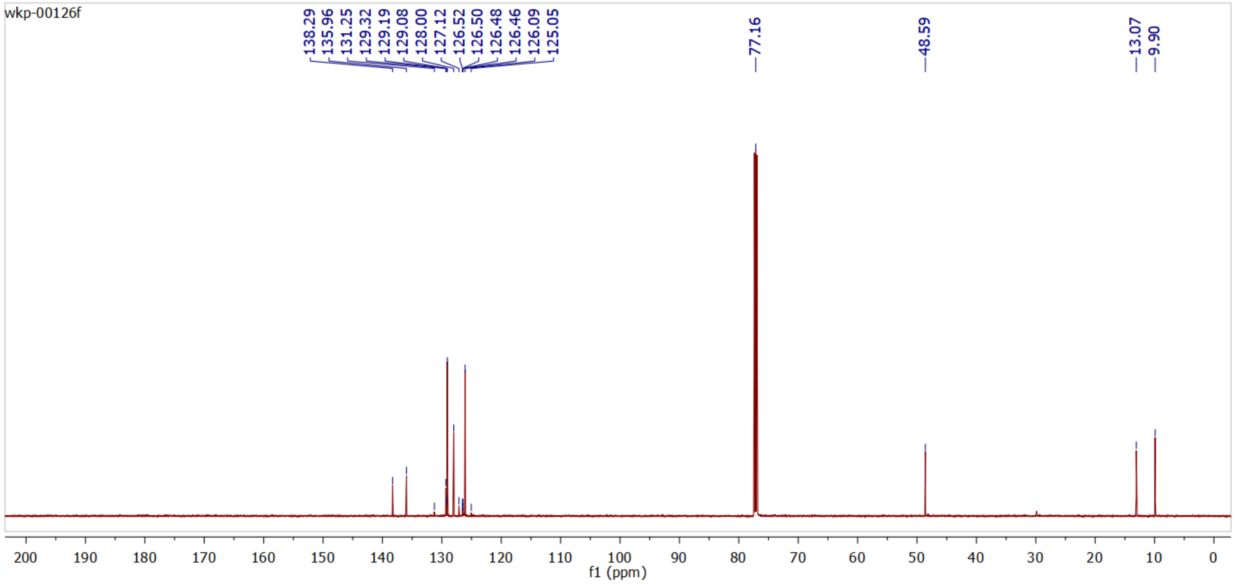

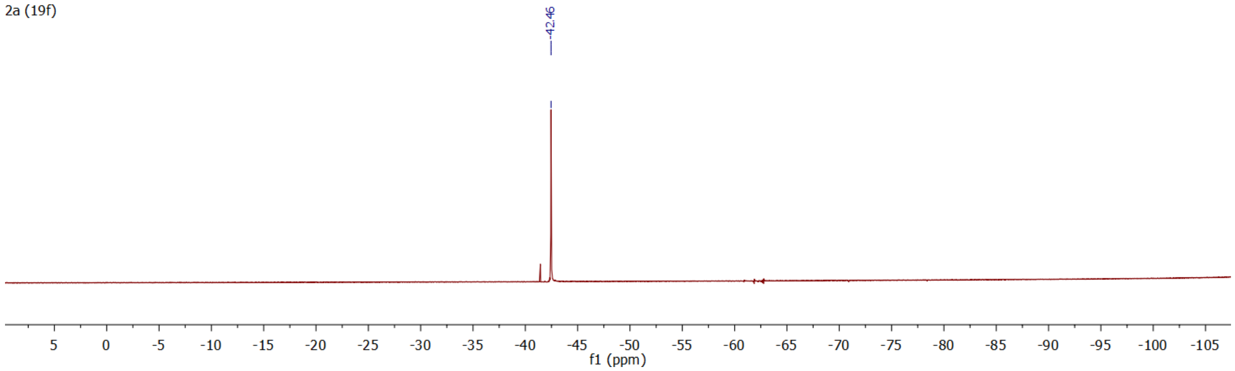

Fig S1. ^{1}H NMR (600 MHz, $CDCl_3$), $^{13}C\{^{1}H\}$ NMR (151 MHz, $CDCl_3$) and ^{19}F NMR (565 MHz, $CDCl_3$) spectra for compound **2a**.

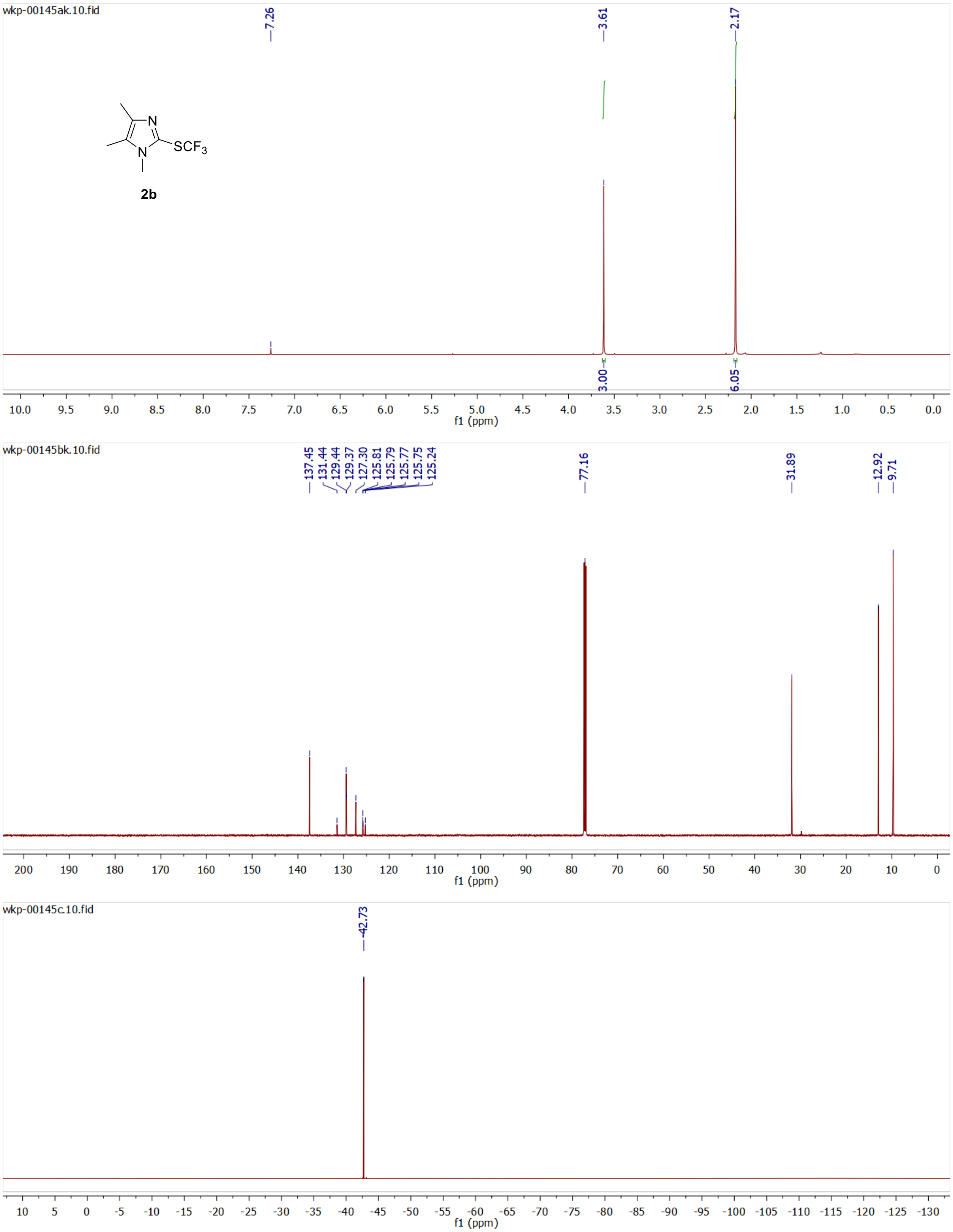

Fig S2. ^{1}H NMR (600 MHz, $CDCl_3$), $^{13}C\{^{1}H\}$ NMR (151 MHz, $CDCl_3$) and ^{19}F NMR (565 MHz, $CDCl_3$) spectra for compound **2b**.

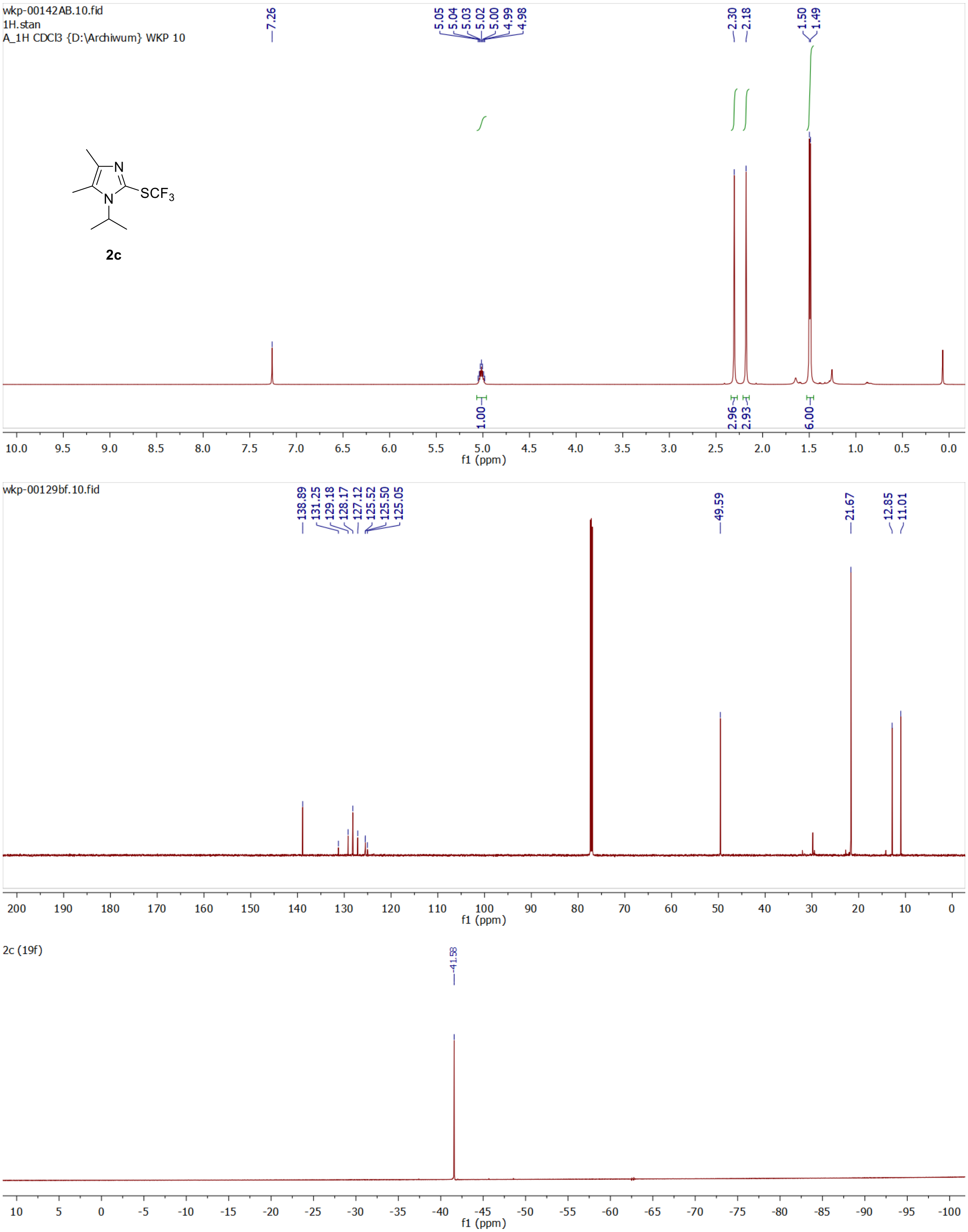

Fig S3. ^{1}H NMR (600 MHz, $CDCl_3$), $^{13}C\{^{1}H\}$ NMR (151 MHz, $CDCl_3$) and ^{19}F NMR (565 MHz, $CDCl_3$) spectra for compound **2c**.

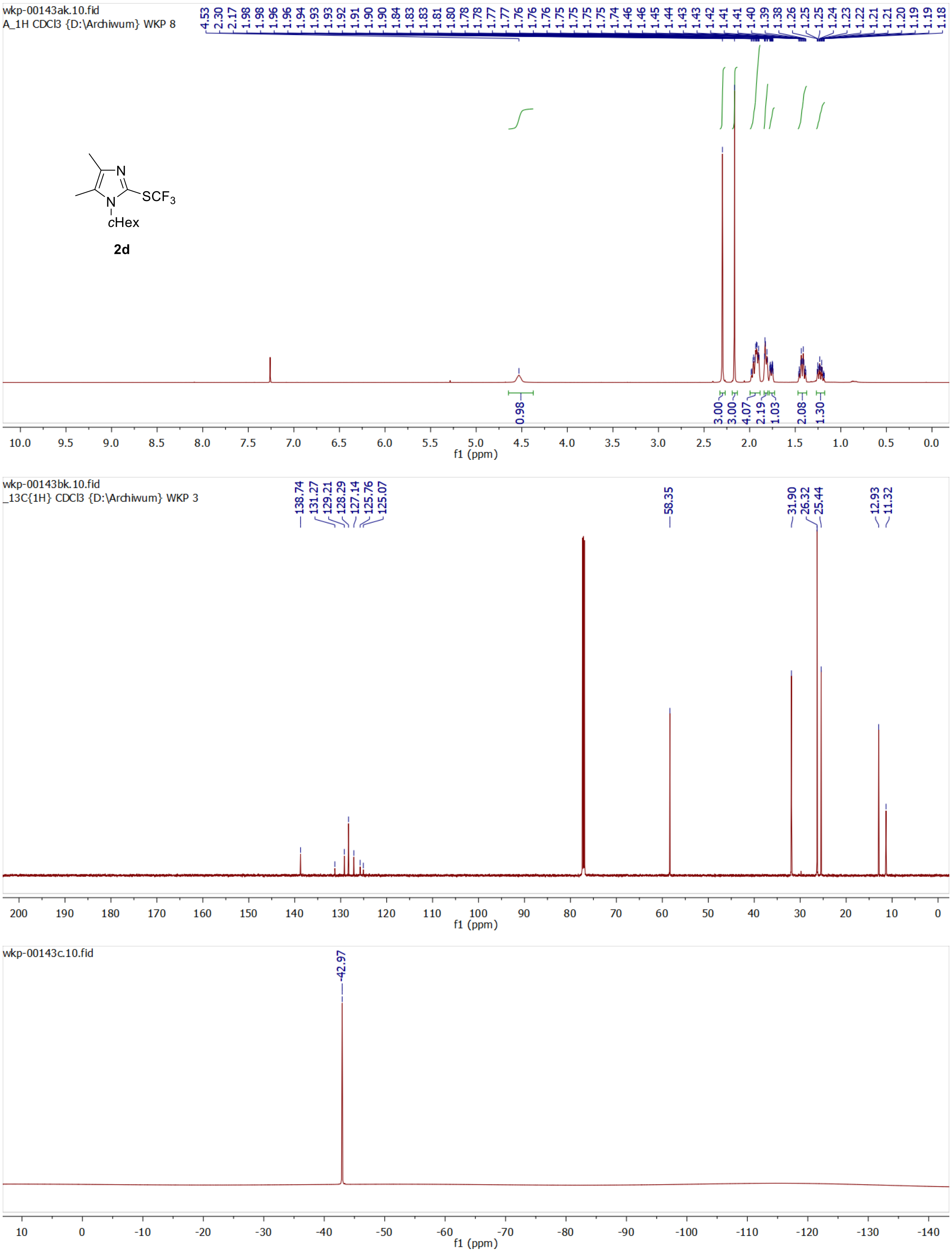

Fig S4. ^{1}H NMR (600 MHz, $CDCl_3$), $^{13}C\{^{1}H\}$ NMR (151 MHz, $CDCl_3$) and ^{19}F NMR (565 MHz, $CDCl_3$) spectra for compound **2d**.

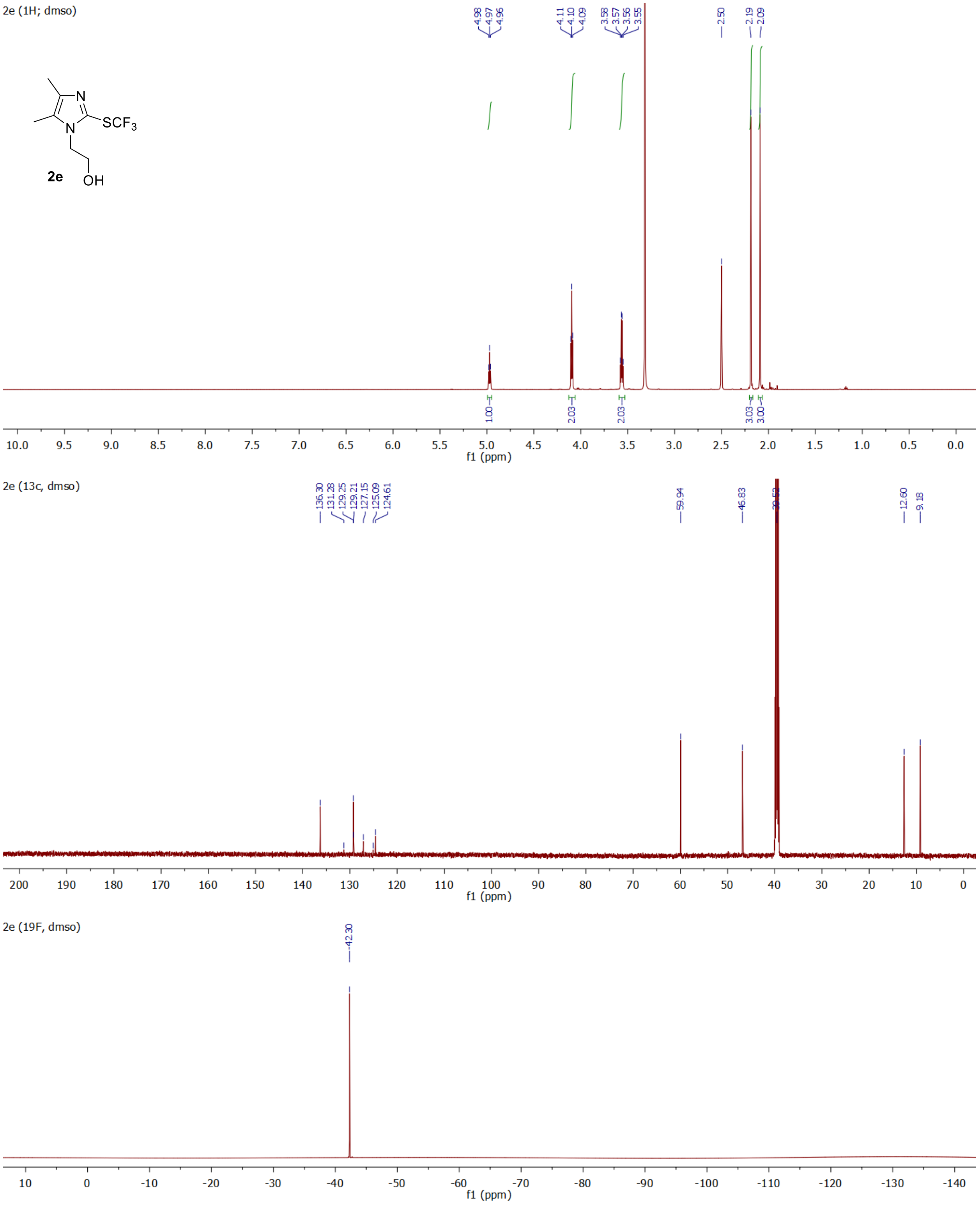

Fig S5. ^{1}H NMR (600 MHz, DMSO-d_6), $^{13}C\{^{1}H\}$ NMR (151 MHz, DMSO-d_6) and ^{19}F NMR (565 MHz, DMSO-d_6) spectra for compound **2e**.

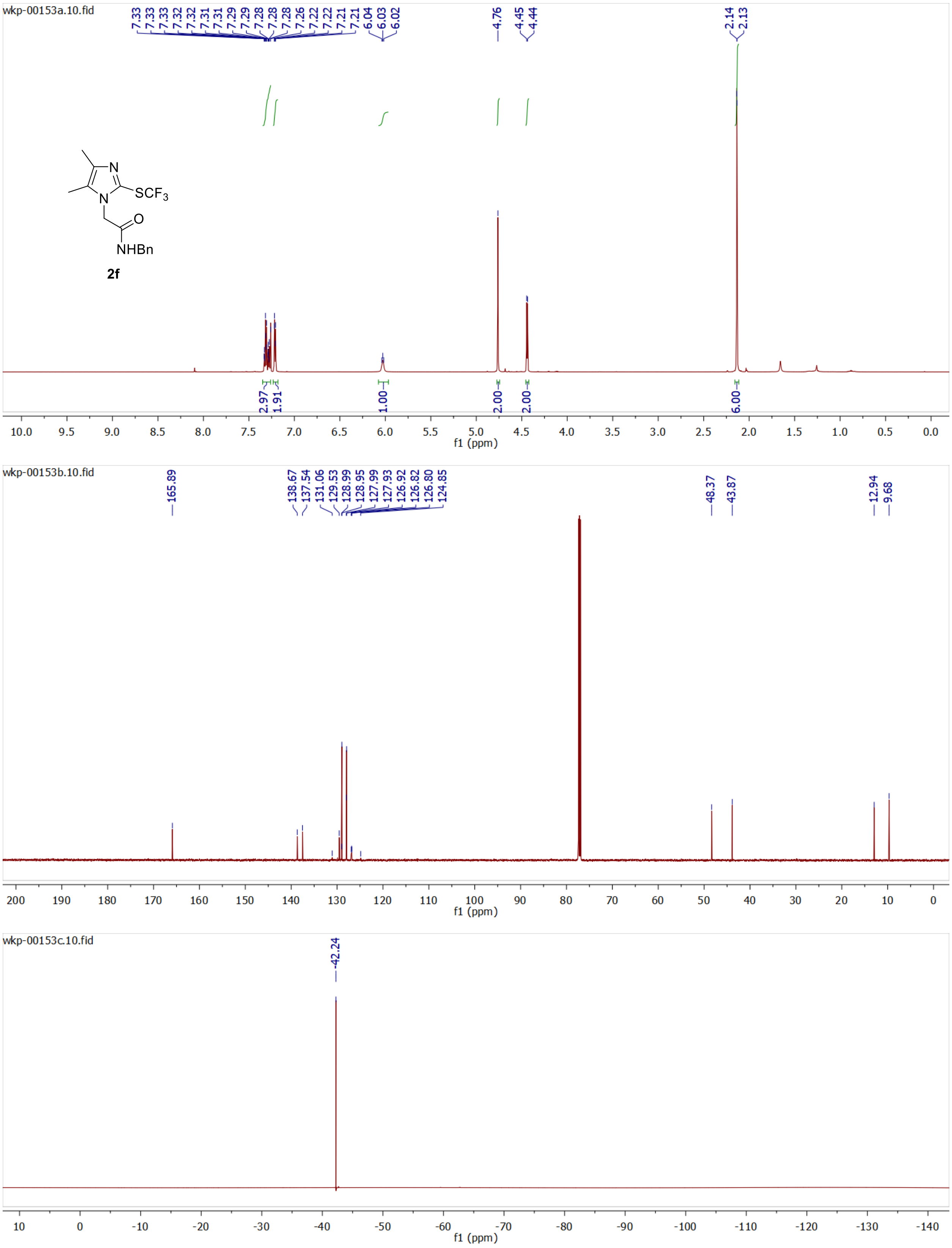

Fig S6. ^{1}H NMR (600 MHz, $CDCl_3$), $^{13}C\{^{1}H\}$ NMR (151 MHz, $CDCl_3$) and ^{19}F NMR (565 MHz, $CDCl_3$) spectra for compound **2f**.

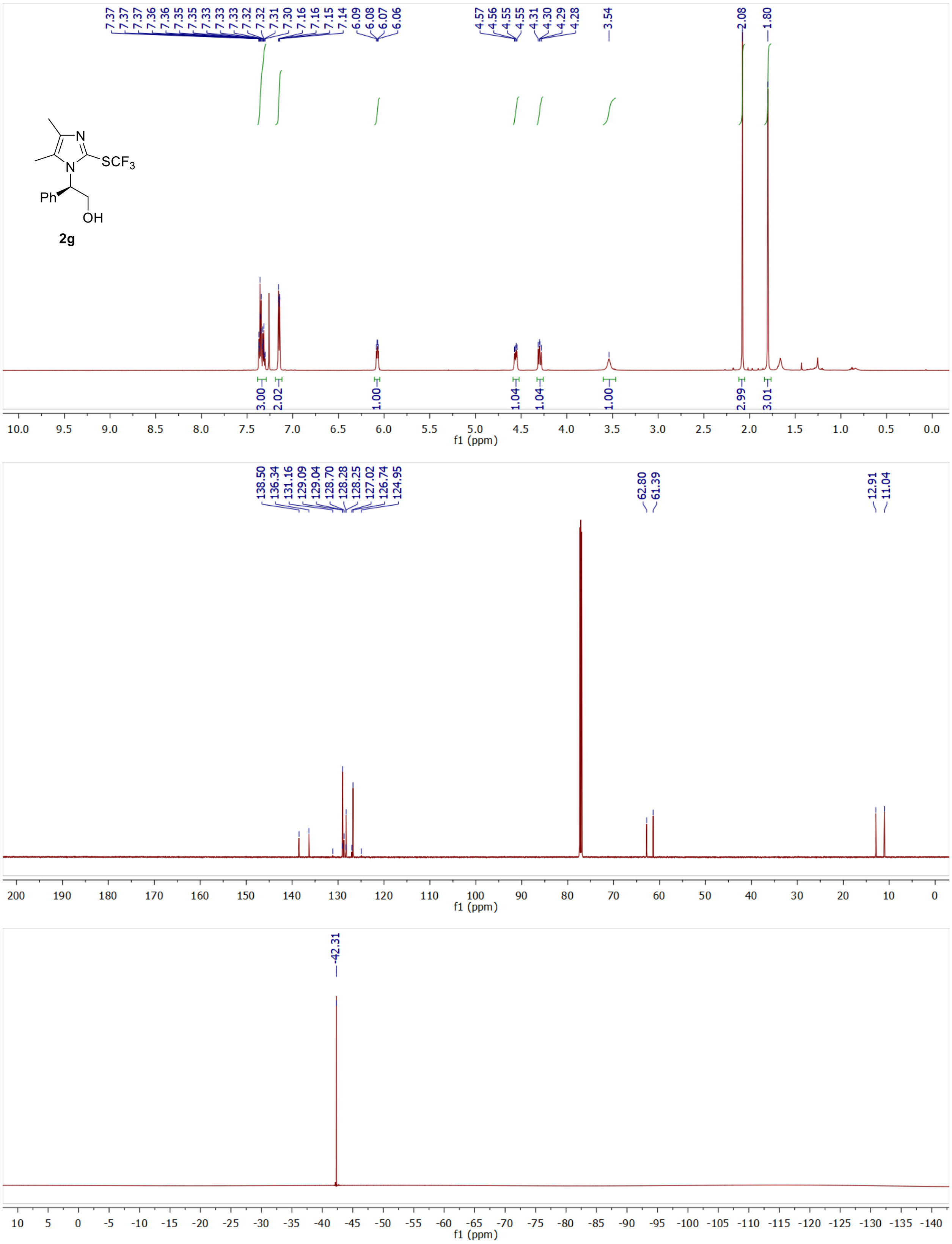

Fig S7. ^1H NMR (600 MHz, $CDCl_3$), $^{13}C\{^1H\}$ NMR (151 MHz, $CDCl_3$) and ^{19}F NMR (565 MHz, $CDCl_3$) spectra for compound **2g**.

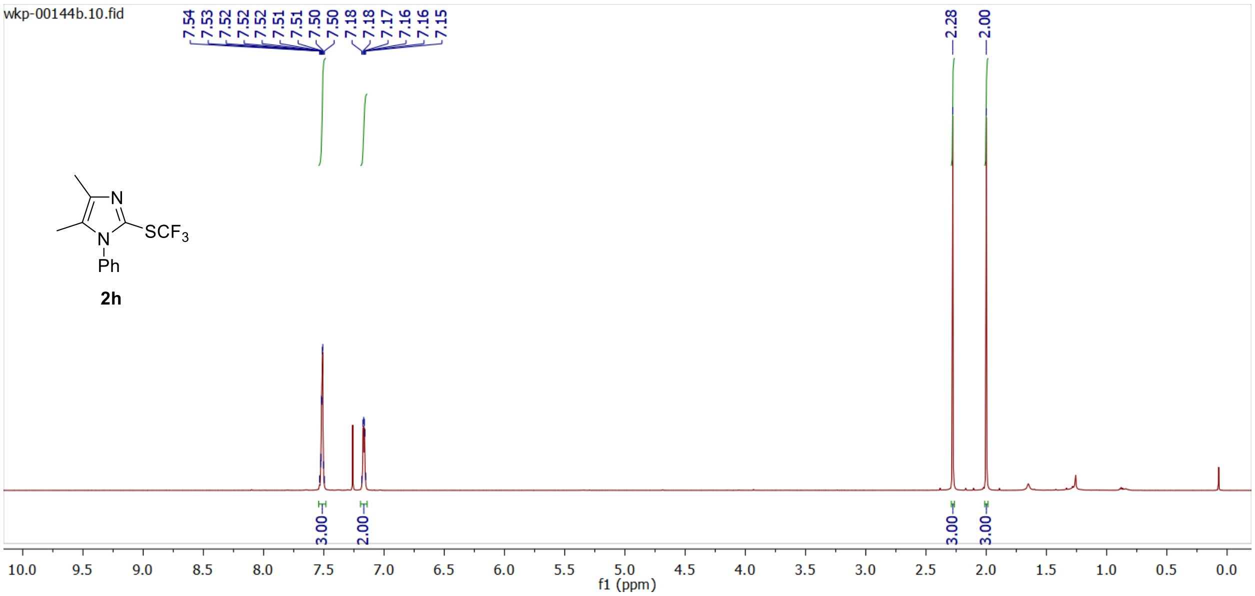

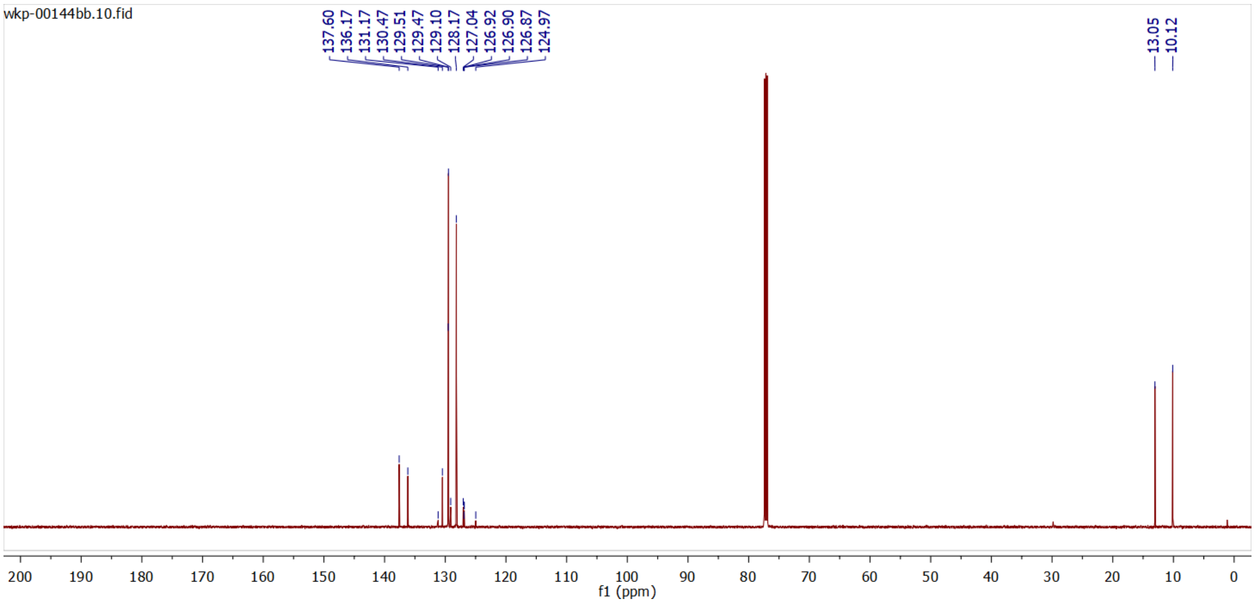

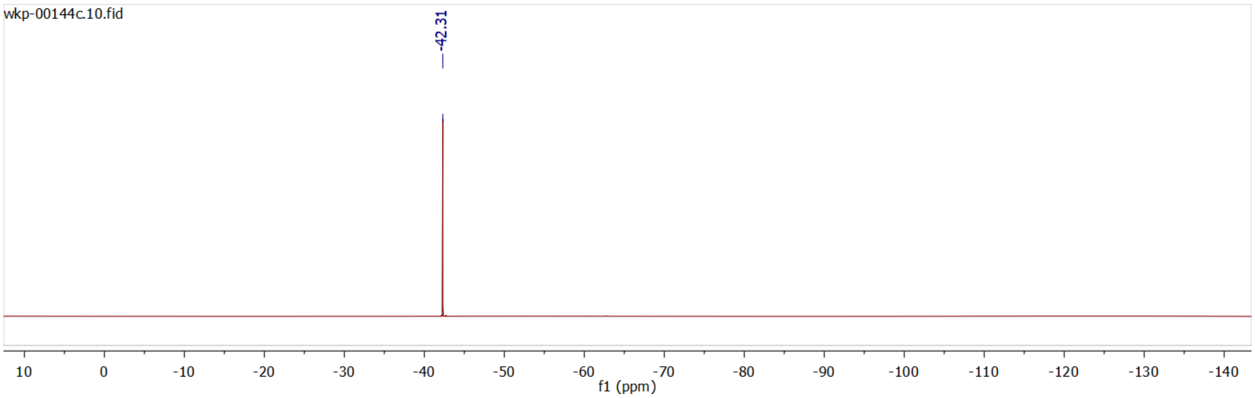

Fig S8. ^{1}H NMR (600 MHz, $CDCl_3$), $^{13}C\{^{1}H\}$ NMR (151 MHz, $CDCl_3$) and ^{19}F NMR (565 MHz, $CDCl_3$) spectra for compound **2h**.

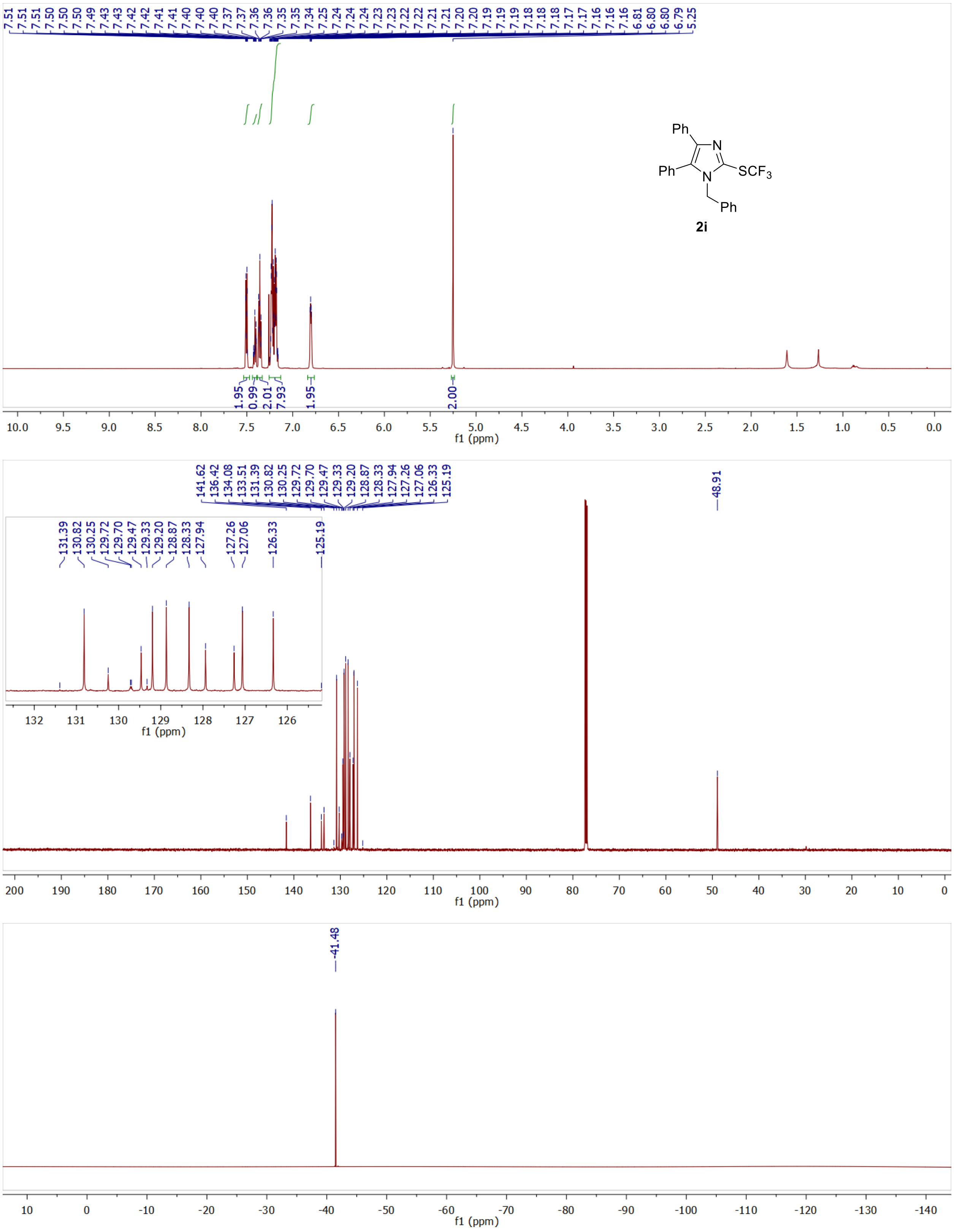

Fig S9. ^{1}H NMR (600 MHz, $CDCl_3$), $^{13}C\{^{1}H\}$ NMR (151 MHz, $CDCl_3$) and ^{19}F NMR (565 MHz, $CDCl_3$) spectra for compound **2i**.

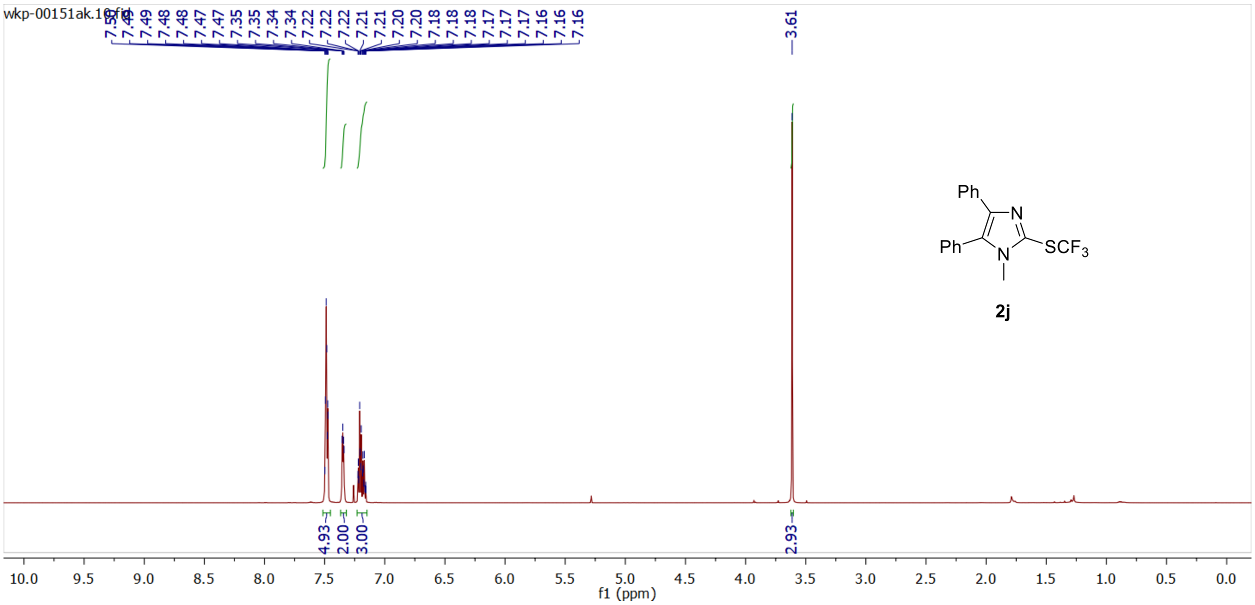

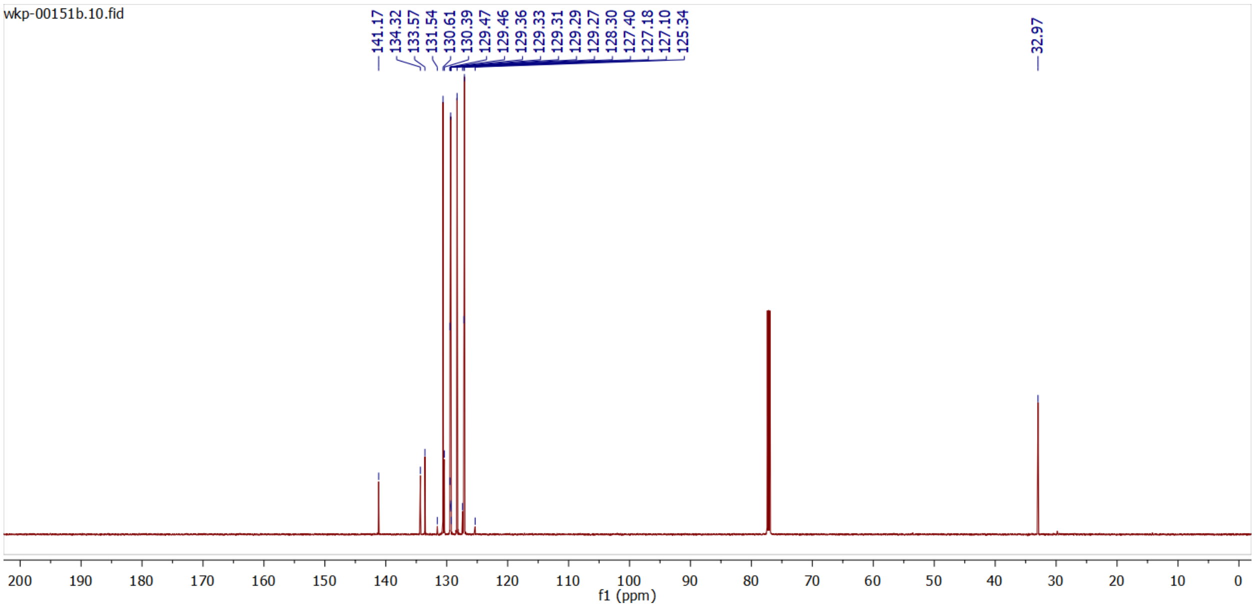

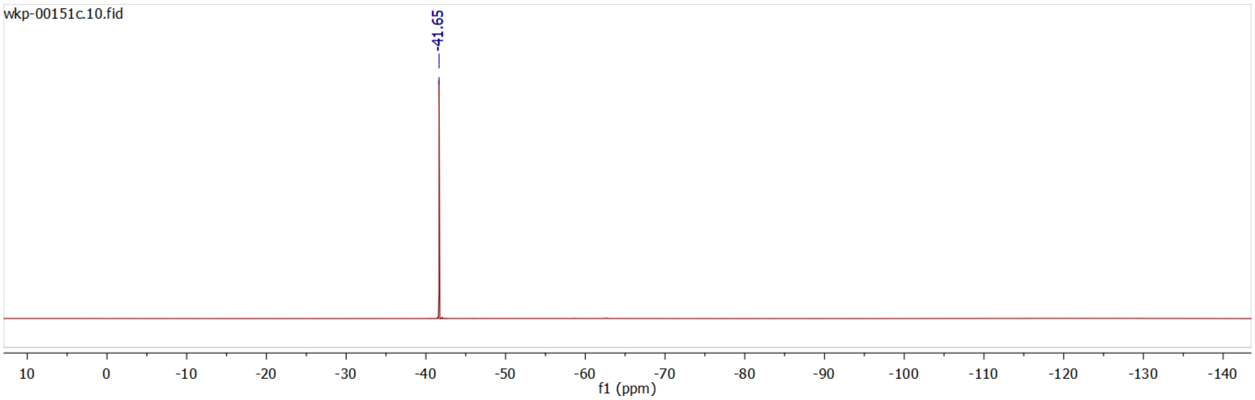

Fig S10. ^{1}H NMR (600 MHz, $CDCl_3$), $^{13}C\{^{1}H\}$ NMR (151 MHz, $CDCl_3$) and ^{19}F NMR (565 MHz, $CDCl_3$) spectra for compound **2j**.

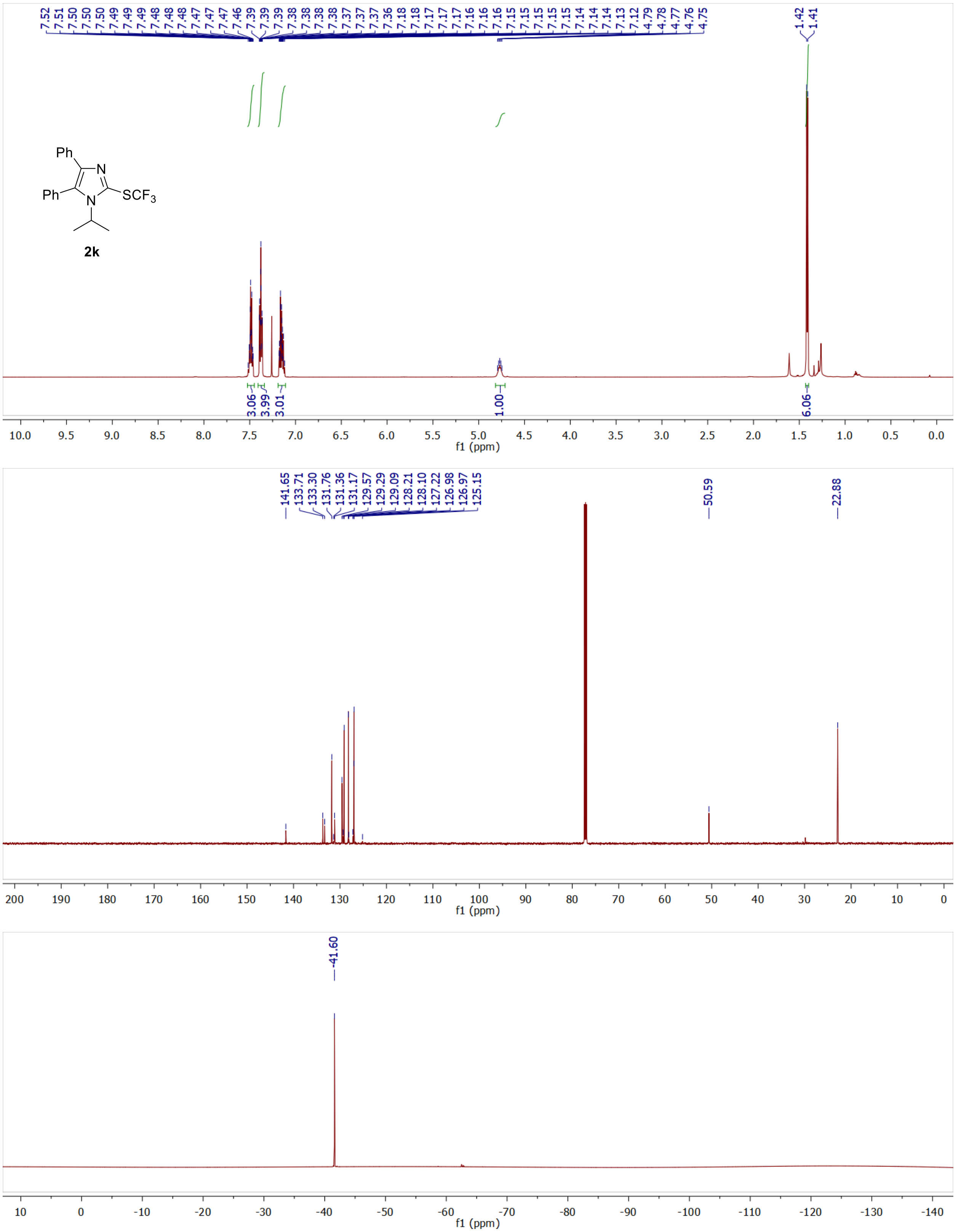

Fig S11. ^{1}H NMR (600 MHz, $CDCl_3$), $^{13}C\{^{1}H\}$ NMR (151 MHz, $CDCl_3$) and ^{19}F NMR (565 MHz, $CDCl_3$) spectra for compound **2k**.

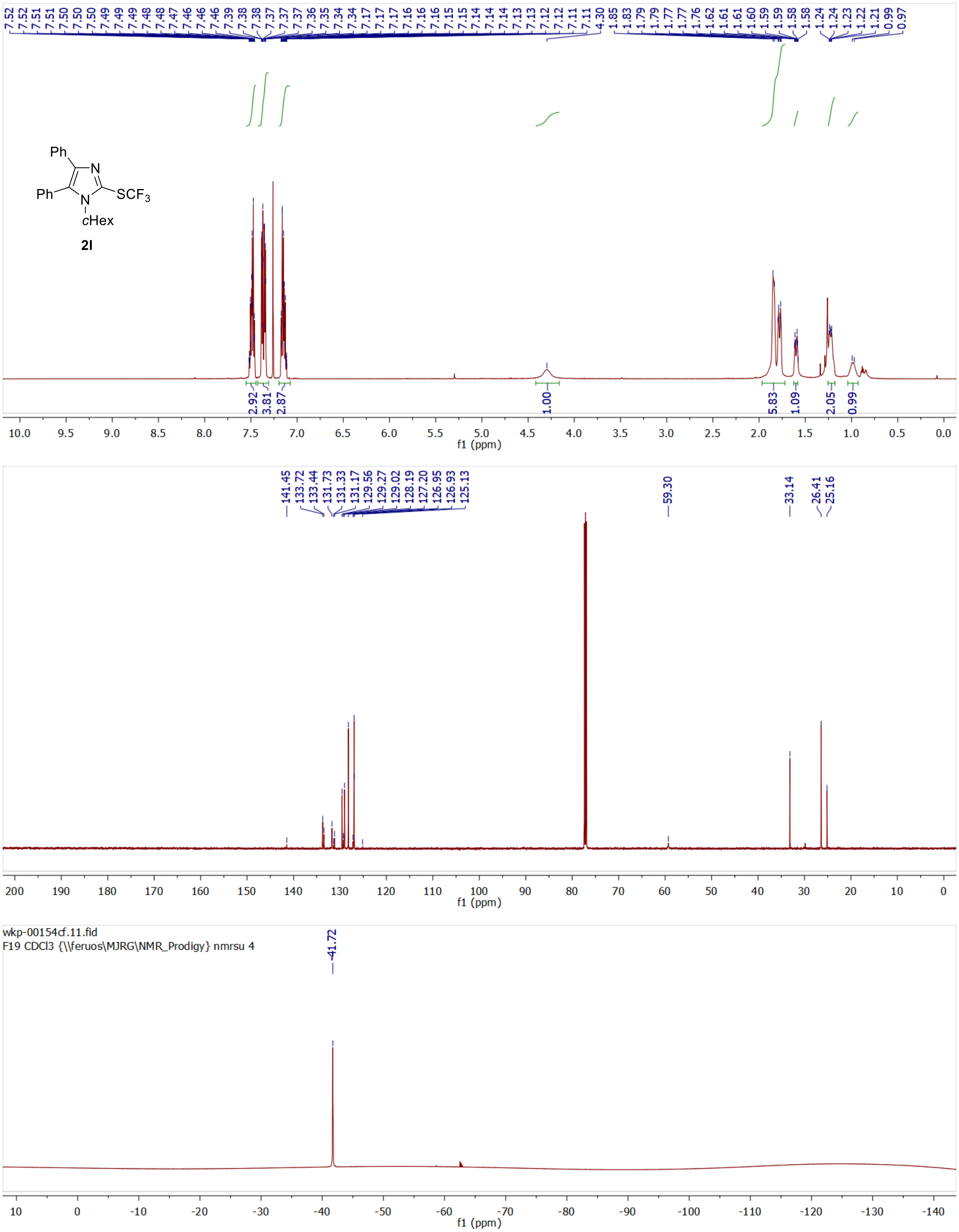

Fig S12. ^{1}H NMR (600 MHz, $CDCl_3$), $^{13}C\{^{1}H\}$ NMR (151 MHz, $CDCl_3$) and ^{19}F NMR (565 MHz, $CDCl_3$) spectra for compound **2l**.

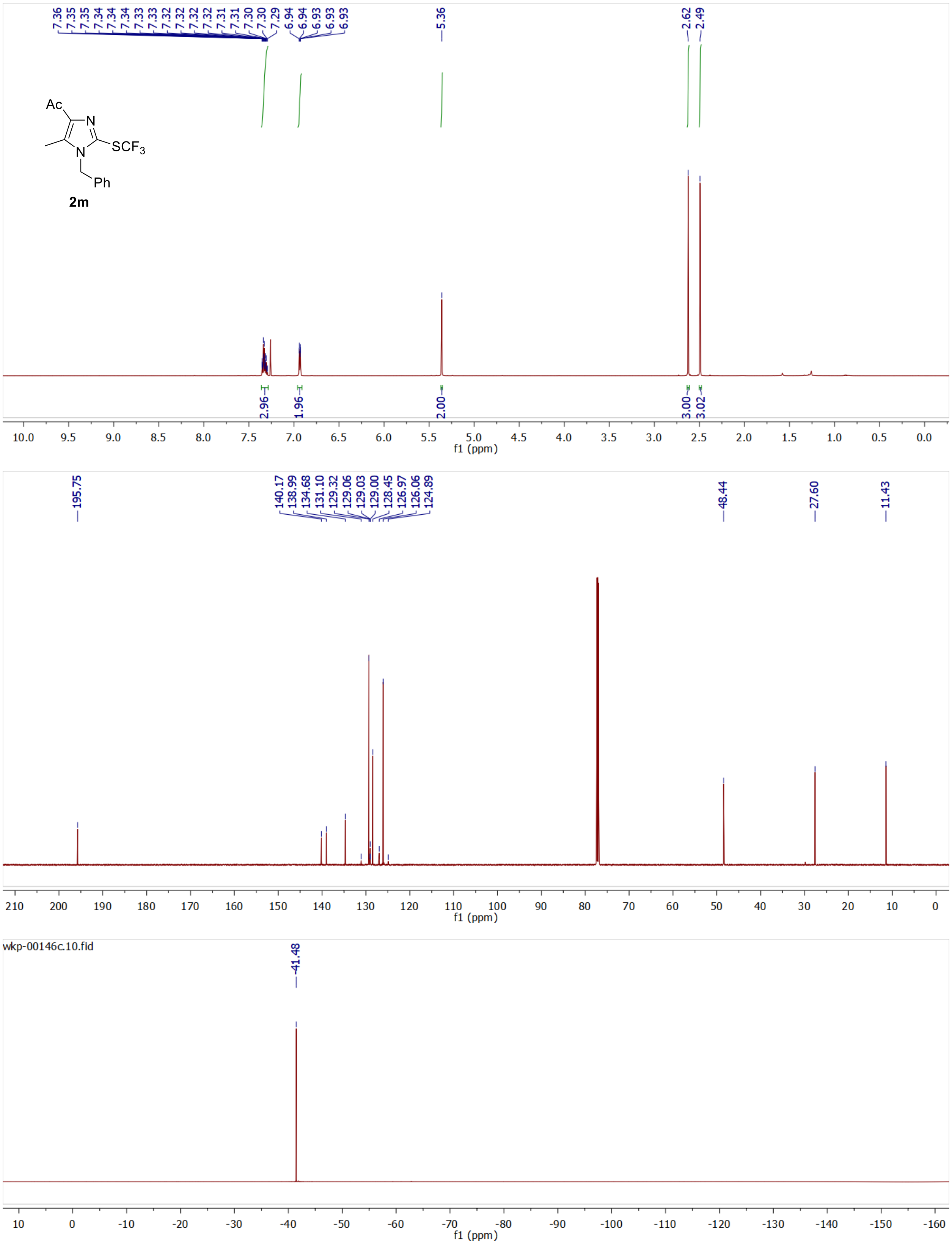

Fig S13. ^{1}H NMR (600 MHz, $CDCl_3$), $^{13}C\{^{1}H\}$ NMR (151 MHz, $CDCl_3$) and ^{19}F NMR (565 MHz, $CDCl_3$) spectra for compound **2m**.

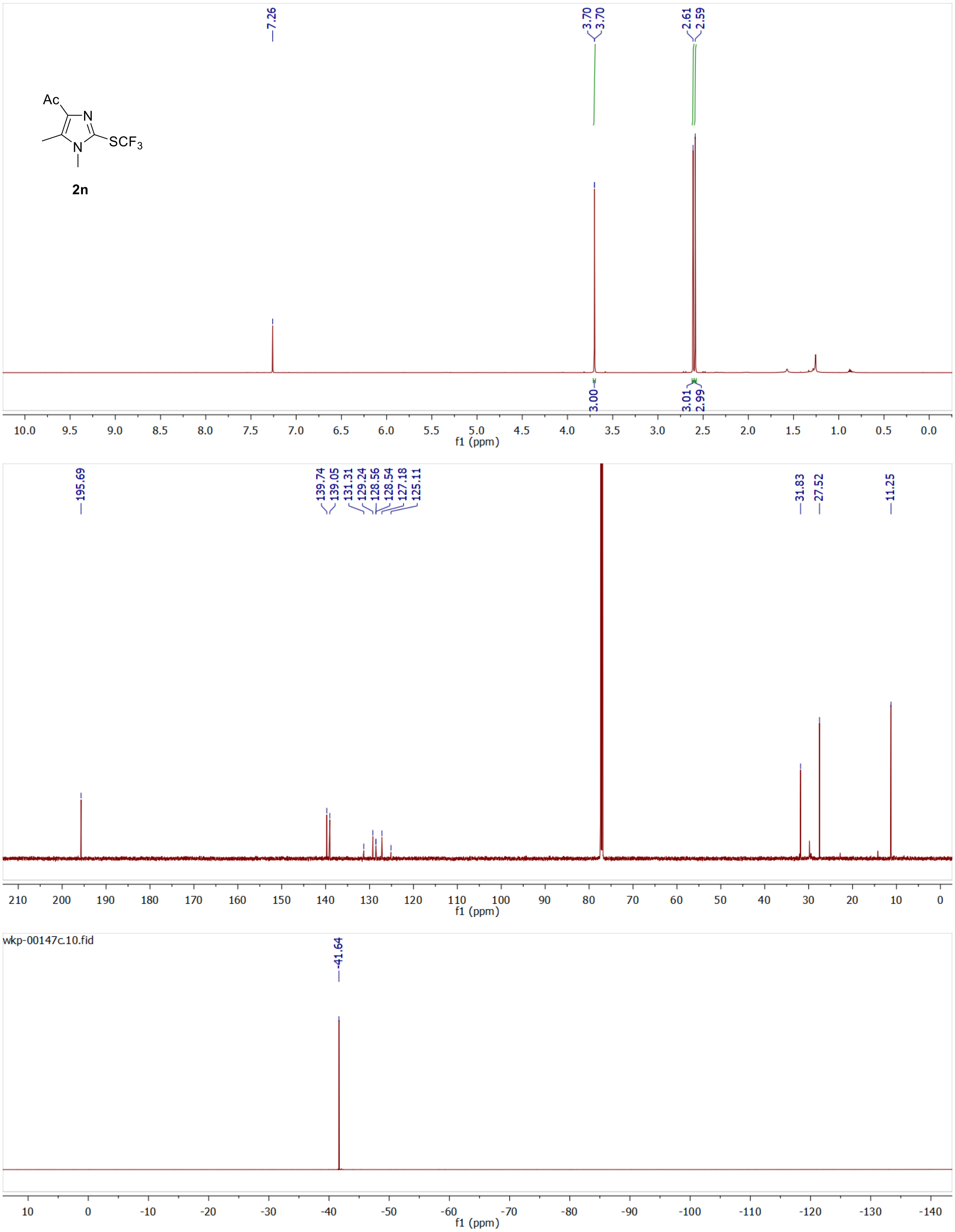

Fig S14. ^{1}H NMR (600 MHz, CDCl$_3$), ^{13}C{^{1}H} NMR (151 MHz, CDCl$_3$) and ^{19}F NMR (565 MHz, CDCl$_3$) spectra for compound **2n**.

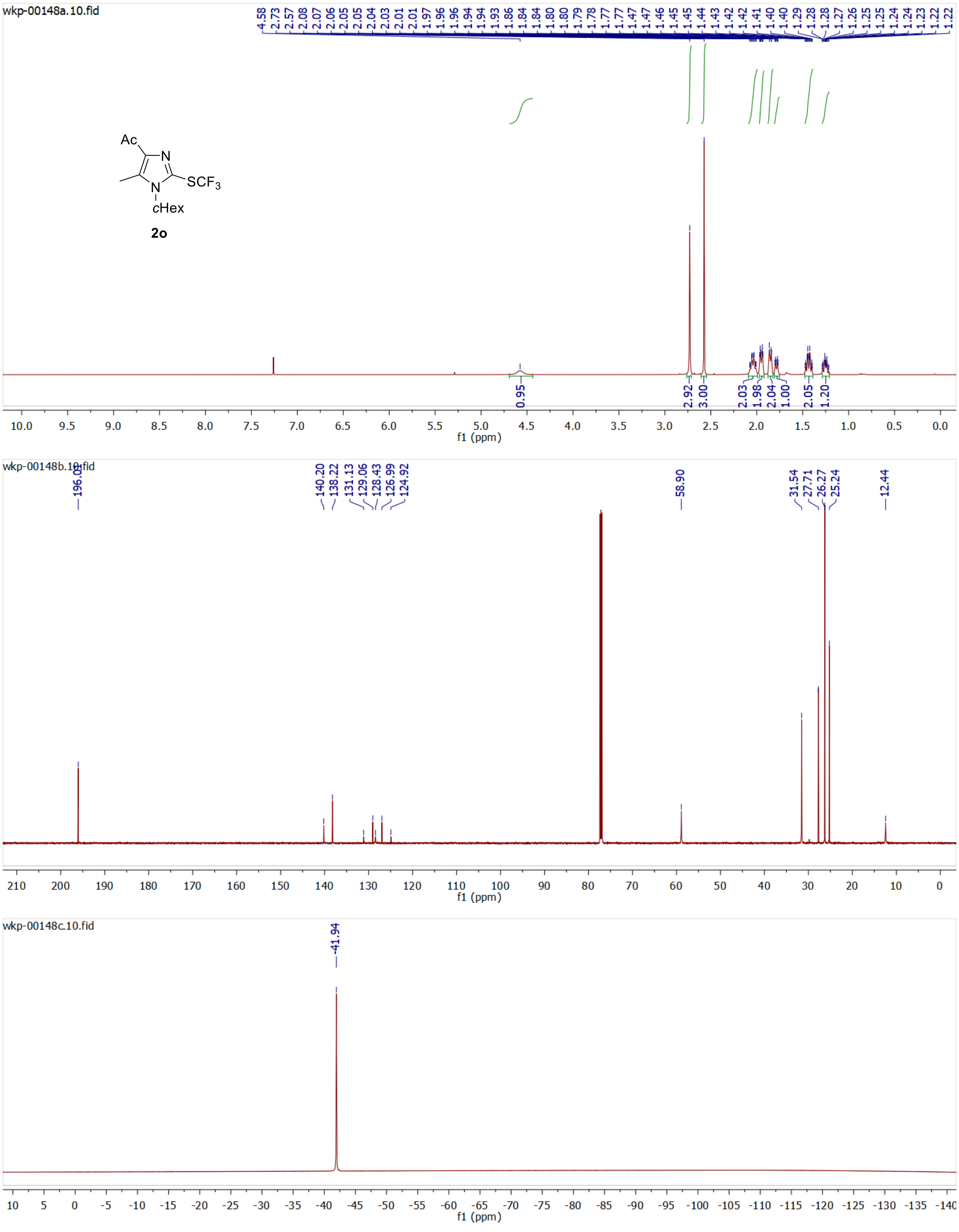

Fig S15. ^{1}H NMR (600 MHz, $CDCl_3$), $^{13}C\{^{1}H\}$ NMR (151 MHz, $CDCl_3$) and ^{19}F NMR (565 MHz, $CDCl_3$) spectra for compound **2o**.

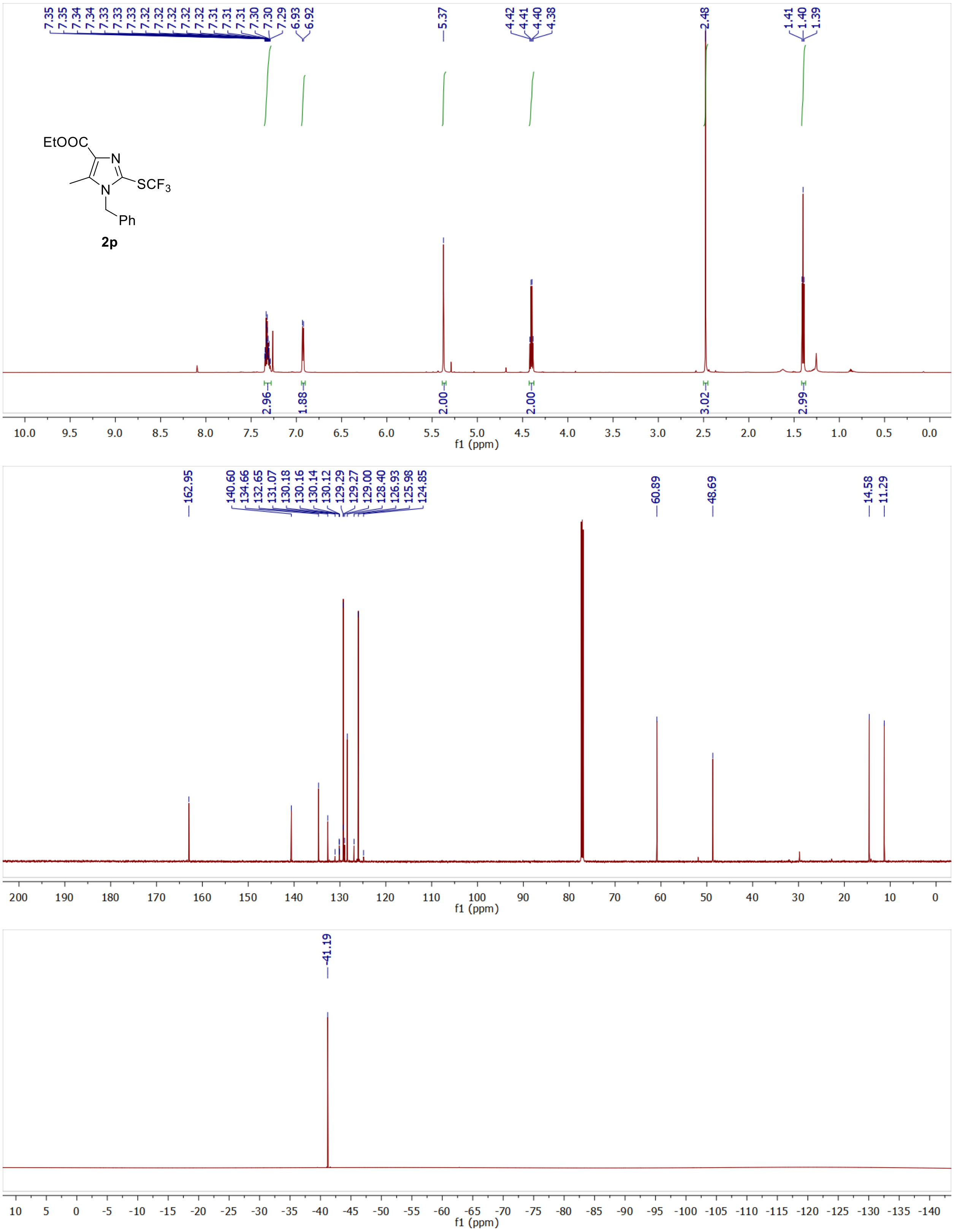

Fig S16. ^{1}H NMR (600 MHz, $CDCl_3$), $^{13}C\{^{1}H\}$ NMR (151 MHz, $CDCl_3$) and ^{19}F NMR (565 MHz, $CDCl_3$) spectra for compound **2p**.

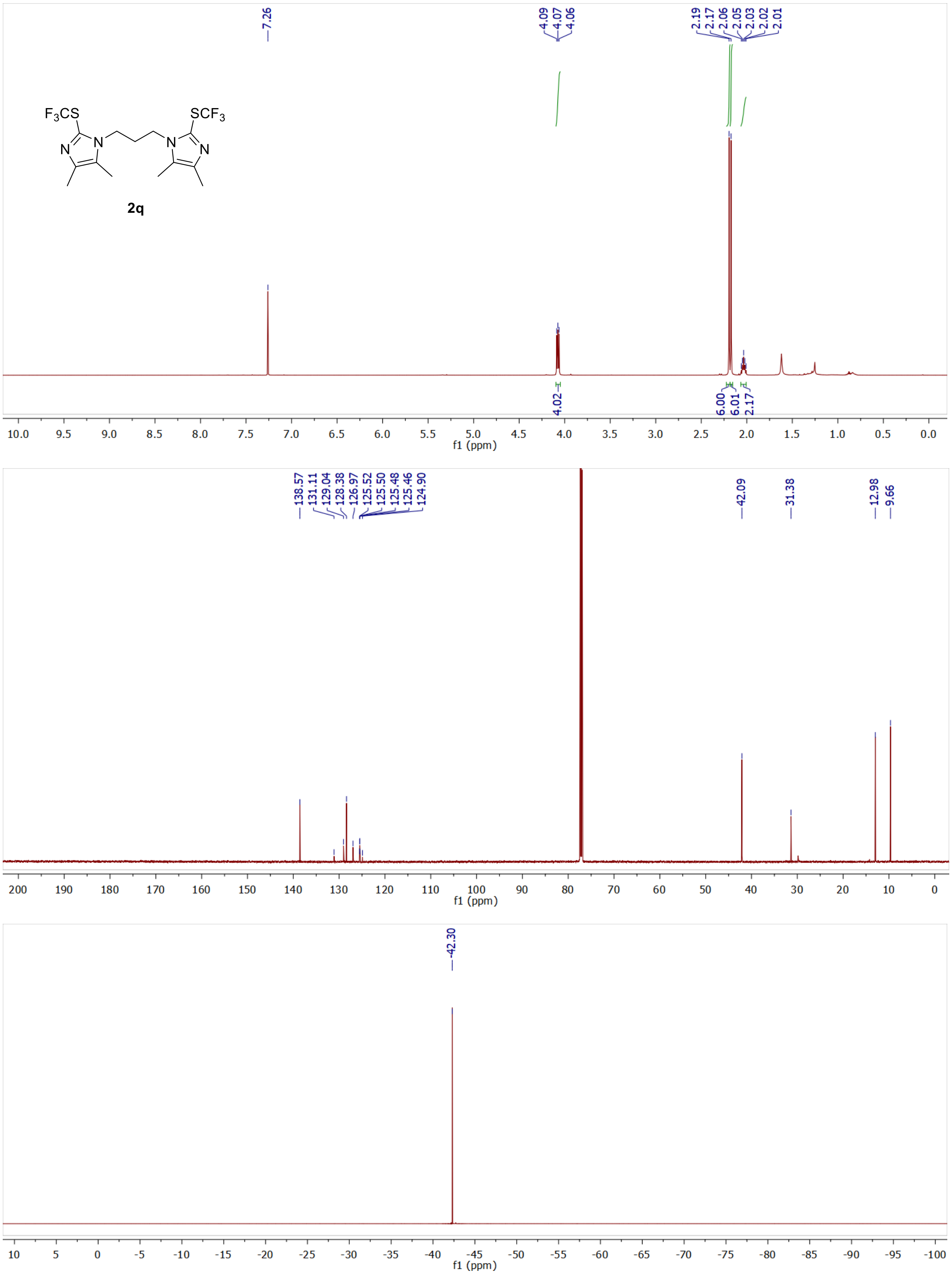

Fig S17. ^{1}H NMR (600 MHz, $CDCl_3$), $^{13}C\{^{1}H\}$ NMR (151 MHz, $CDCl_3$) and ^{19}F NMR (565 MHz, $CDCl_3$) spectra for compound **2q**.

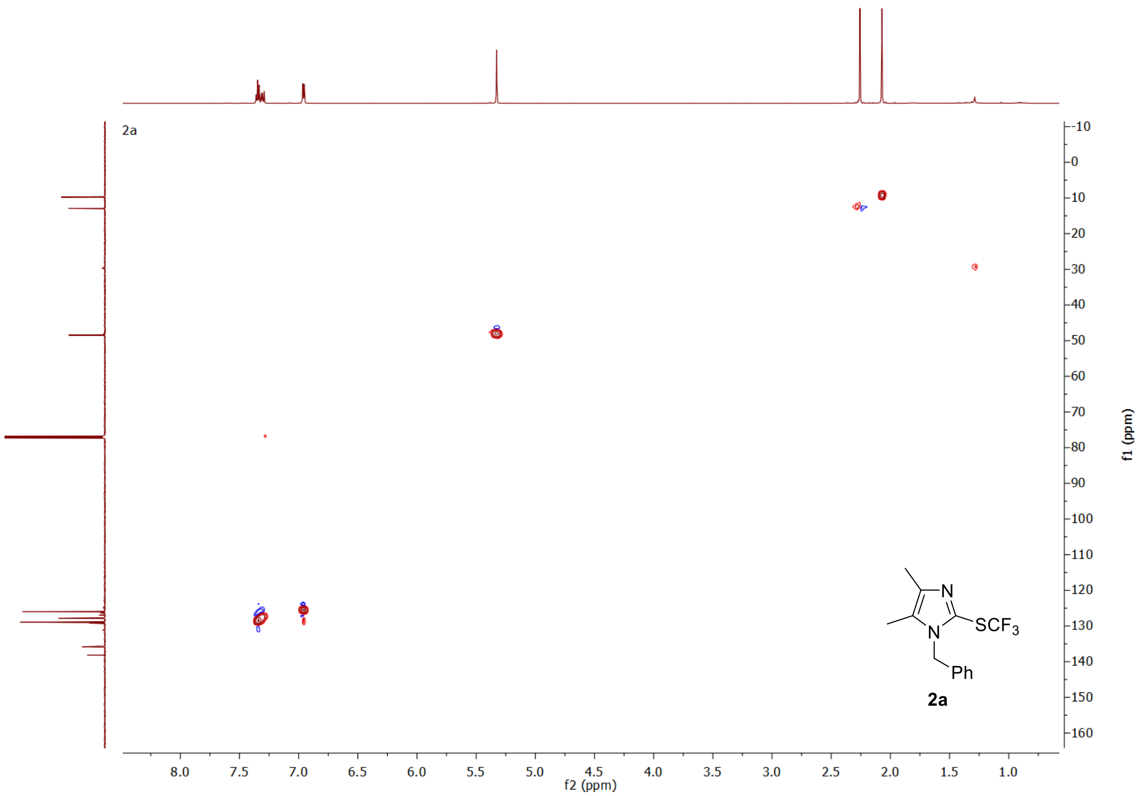

Fig S18. HMQC ($CDCl_3$) for **2a**.

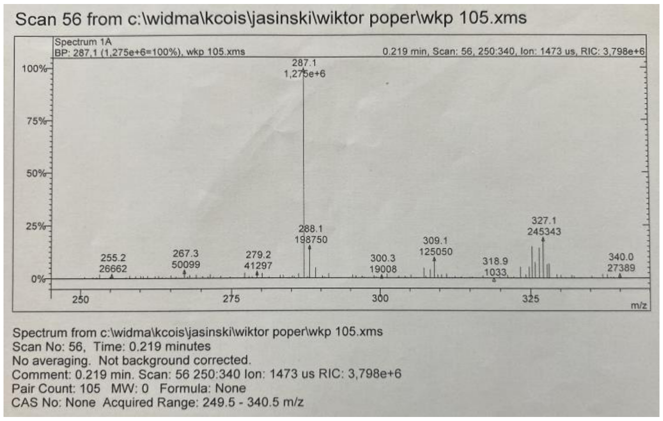

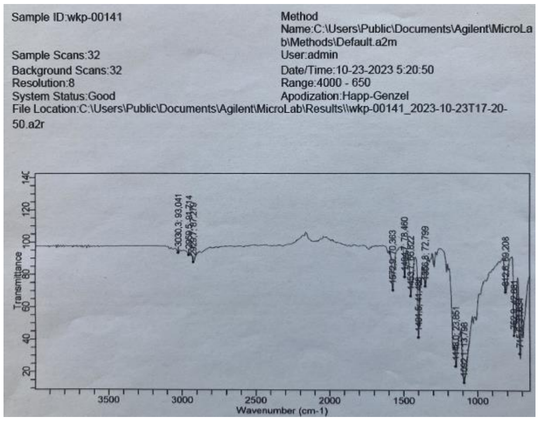

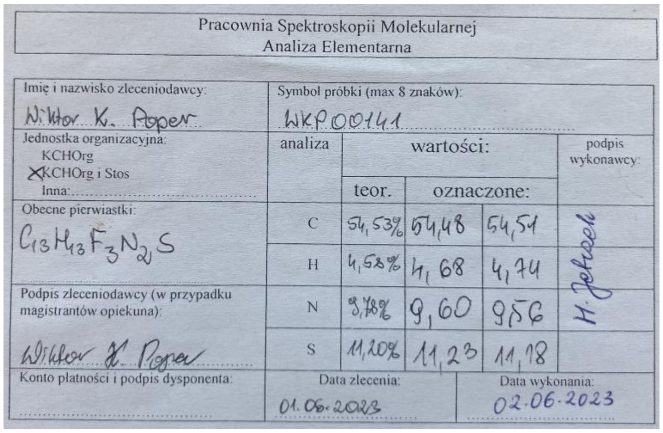

Pracownia Spektroskopii Molekularnej
Analiza Elementarna

Imię i nazwisko zleceniodawcy: Wiktor K. Poper	Symbol próbki (max 8 znaków): WKP00141			
Jednostka organizacyjna: KCHOrg; X KCHOrg i Stos; Inna:	analiza	wartości: teor.	oznaczone:	oznaczone:
Obecne pierwiastki: $C_{13}H_{13}F_3N_2S$	C	54,53%	54,48	54,51
	H	4,58%	4,68	4,74
Podpis zleceniodawcy (w przypadku magistrantów opiekuna): Wiktor K. Poper	N	9,78%	9,60	9,56
	S	11,20%	11,23	11,18
Konto płatności i podpis dysponenta:	Data zlecenia: 01.06.2023		Data wykonania: 02.06.2023	

podpis wykonawcy: H. Jeleń

Fig S19. (+)-ESI-MS, IR, and EA analyses for **2a**.

4. Crystallographic analysis

Crystallographic analysis of 2g: Single crystals of **2g** ($C_{14}H_{15}F_3N_2OS$) were measured. A suitable crystal was selected and measured on a XtaLAB Synergy, Dualflex, Pilatus 300K diffractometer. The crystal was kept at 100 K during data collection. Using Olex2,[3a] the structure was solved with the SHELXT[3b] structure solution program using Intrinsic Phasing and refined with the XL[3c] refinement package using Least Squares minimization. Crystallographic data have been deposited at the Cambridge Crystallographic Data Center as supplementary publication numbers CCDC-2286607. These data can be obtained free of charge from the Cambridge Crystallographic Data Centre via https://www.ccdc.cam.ac.uk/structures/

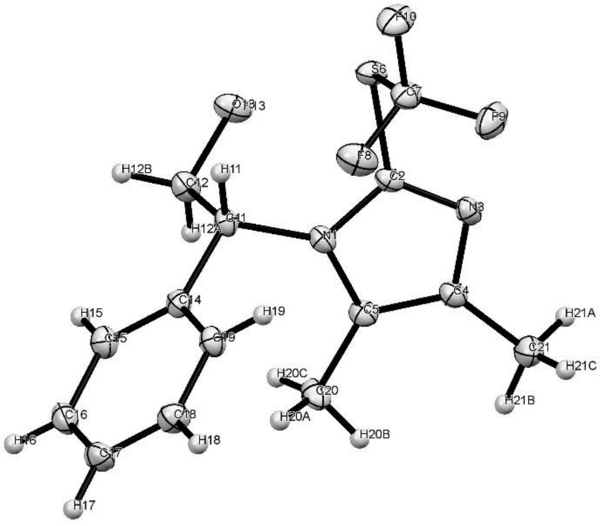

Fig S20. A view of the molecular structure of compound **2g**. Displacement ellipsoids are drawn at the 50% probability level. X-ray data collected at the ambient temperature 100 K.

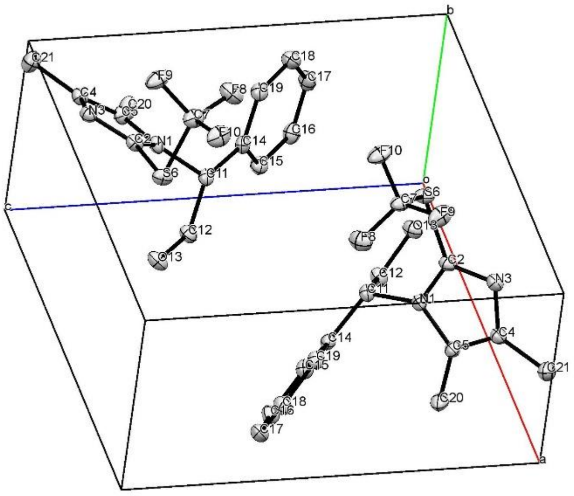

Fig S21. A view of the molecular packing in the structure of compound **2g**.

Crystal structure determination of 2g: Crystal Data for $C_{14}H_{15}F_3N_2OS$ (*M* =316.34 g/mol): monoclinic, space group $P2_1$ (no. 4), *a* = 8.02196(5) Å, *b* = 9.17291(7) Å, *c* = 10.23087(6) Å, *β* = 104.7965(6)°, *V* = 727.870(9) Å^3, *Z* = 2, *T* = 100 K, μ(Cu Kα) = 2.303 mm^{-1}, *Dcalc* = 1.443 g/cm^3, 47326 reflections measured (8.94° ≤ 2Θ ≤ 159.76°), 2995 unique (R_{int} = 0.0328, R_{sigma} = 0.0111) which were used in all calculations. The final R_1 was 0.0192 (I > 2σ(I)) and wR_2 was 0.0512 (all data).

Refinement model description
Number of restraints - 1, number of constraints - unknown.

Details:
1. Fixed Uiso
 At 1.2 times of:
  All C(H) groups, All C(H,H) groups
 At 1.5 times of:
  All C(H,H,H) groups, All O(H) groups
2.a Ternary CH refined with riding coordinates:
 C11(H11)
2.b Secondary CH2 refined with riding coordinates:
 C12(H12A,H12B)
2.c Aromatic/amide H refined with riding coordinates:
 C19(H19), C15(H15), C17(H17), C18(H18), C16(H16)
2.d Idealised Me refined as rotating group:
 C20(H20A,H20B,H20C), C21(H21A,H21B,H21C)
2.e Idealised tetrahedral OH refined as rotating group:
 O13(H13)

Table S2. Crystal data and structure refinement for **2g**.

Identification code	158_2
Empirical formula	$C_{14}H_{15}F_3N_2OS$
Formula weight	316.34
Temperature/K	100
Crystal system	monoclinic
Space group	$P2_1$
a/Å	8.02196(5)
b/Å	9.17291(7)
c/Å	10.23087(6)
α/°	90
β/°	104.7965(6)
γ/°	90
Volume/Å^3	727.870(9)
Z	2
ρ_{calc}g/cm^3	1.443
μ/mm^{-1}	2.303
F(000)	328.0
Crystal size/mm^3	0.88 × 0.14 × 0.08
Radiation	Cu Kα (λ = 1.54184)
2Θ range for data collection/°	8.94 to 159.76

Index ranges	-9 ≤ h ≤ 10, -11 ≤ k ≤ 10, -13 ≤ l ≤ 12
Reflections collected	47326
Independent reflections	2995 [R_{int} = 0.0328, R_{sigma} = 0.0111]
Data/restraints/parameters	2995/1/194
Goodness-of-fit on F^2	1.080
Final R indexes [I>=2σ (I)]	R_1 = 0.0192, wR_2 = 0.0512
Final R indexes [all data]	R_1 = 0.0193, wR_2 = 0.0512
Largest diff. peak/hole / e Å^{-3}	0.17/-0.14
Flack parameter	0.009(4)

Table S3. Fractional Atomic Coordinates (×10^4) and Equivalent Isotropic Displacement Parameters (Å^2×10^3) for **2g**. U_{eq} is defined as 1/3 of the trace of the orthogonalised U_{IJ} tensor.

Atom	*x*	*y*	*z*	U(eq)
S6	4163.9(5)	6352.2(5)	1496.0(4)	18.75(11)
F8	6250.8(14)	7507.2(13)	3662.0(11)	29.4(3)
F9	6236.2(14)	8640.2(12)	1826.4(12)	29.0(3)
F10	4034.7(13)	8791.0(11)	2666.9(11)	27.8(2)
O13	3277.6(15)	2895.3(14)	1328.7(12)	21.6(3)
N3	6926.8(17)	5657.4(16)	537.6(13)	17.2(3)
N1	6577.2(18)	4160.2(16)	2158.7(13)	15.5(3)
C5	7981(2)	3652.0(19)	1731.0(16)	16.9(3)
C7	5239(2)	7886.5(19)	2449.7(18)	20.8(3)
C14	7064(2)	3012.6(19)	4411.8(16)	16.6(3)
C4	8177(2)	4595.5(19)	742.0(17)	17.4(3)
C19	8269(2)	4031.8(19)	5088.6(16)	19.1(3)
C2	5992(2)	5363.9(17)	1393.8(16)	15.9(3)
C15	7138(2)	1592.9(19)	4907.8(17)	20.2(4)
C17	9620(2)	2210(2)	6716.2(17)	23.5(4)
C12	4503(2)	2287.7(19)	2442.3(16)	19.4(3)
C20	8998(2)	2314(2)	2248.0(18)	23.9(4)
C11	5734(2)	3486.3(18)	3136.9(15)	15.9(3)
C18	9544(2)	3627(2)	6224.6(17)	22.6(4)
C21	9509(2)	4590(2)	-46.5(19)	25.7(4)
C16	8410(2)	1202(2)	6060.7(16)	22.9(3)

Table S4. Anisotropic Displacement Parameters (Å^2×10^3) for **2g**. The Anisotropic displacement factor exponent takes the form: $-2\pi^2[h^2a^{*2}U_{11}+2hka^*b^*U_{12}+\ldots]$.

Atom	U11	U22	U33	U23	U13	U12
S6	15.63(18)	15.76(18)	23.30(19)	-0.40(17)	2.13(13)	2.06(15)
F8	29.2(5)	27.3(6)	27.2(5)	-5.2(4)	-0.9(4)	1.8(5)
F9	28.8(5)	19.2(5)	43.7(6)	-0.7(5)	18.1(5)	-3.8(4)

F10	27.2(5)	17.2(5)	42.2(6)	-3.7(5)	14.8(5)	3.8(4)
O13	22.6(6)	19.7(6)	18.5(6)	-4.7(5)	-1.9(4)	1.6(5)
N3	18.3(7)	16.7(7)	15.2(6)	0.7(5)	1.8(5)	-0.8(5)
N1	19.0(7)	13.4(7)	13.6(6)	-0.6(5)	3.3(5)	1.3(5)
C5	19.2(7)	15.6(8)	15.9(7)	-1.6(6)	4.6(6)	2.5(6)
C7	20.7(8)	14.9(8)	27.5(9)	-0.1(7)	7.3(7)	2.5(7)
C14	18.7(7)	17.8(8)	14.2(7)	-0.5(6)	5.9(6)	1.1(6)
C4	18.7(8)	16.9(8)	15.6(7)	-0.1(6)	2.8(6)	1.1(6)
C19	22.5(8)	16.8(8)	18.4(7)	0.0(6)	6.0(6)	-0.1(7)
C2	16.9(7)	13.8(8)	15.3(7)	-0.9(6)	1.2(6)	1.5(6)
C15	23.3(8)	18.3(10)	19.3(7)	-0.7(6)	6.1(6)	-1.4(6)
C17	21.3(8)	30.9(10)	17.1(8)	3.5(7)	2.5(6)	3.8(7)
C12	22.1(8)	17.0(8)	17.6(7)	-0.2(6)	2.4(6)	-1.2(6)
C20	30.3(9)	20.4(9)	23.8(8)	4.4(7)	12.0(7)	9.1(7)
C11	20.0(8)	14.1(8)	14.0(7)	-0.1(6)	4.8(6)	-0.5(6)
C18	21.0(8)	25.1(9)	20.5(8)	-2.3(7)	3.0(6)	-2.2(7)
C21	25.3(9)	29.0(10)	24.8(8)	6.4(8)	10.2(8)	4.8(7)
C16	27.6(8)	21.3(9)	21.1(8)	5.3(7)	8.4(6)	3.2(8)

Table S5. Bond Lengths for **2g**.

Atom	Atom	Length/Å	Atom	Atom	Length/Å
S6	C7	1.8019(18)	C5	C4	1.371(2)
S6	C2	1.7499(16)	C5	C20	1.494(2)
F8	C7	1.343(2)	C14	C19	1.395(2)
F9	C7	1.336(2)	C14	C15	1.393(2)
F10	C7	1.335(2)	C14	C11	1.522(2)
O13	C12	1.415(2)	C4	C21	1.495(2)
N3	C4	1.375(2)	C19	C18	1.388(2)
N3	C2	1.318(2)	C15	C16	1.395(2)
N1	C5	1.389(2)	C17	C18	1.390(3)
N1	C2	1.365(2)	C17	C16	1.384(3)
N1	C11	1.479(2)	C12	C11	1.526(2)

Table S6. Bond Angles for **2g**.

Atom	Atom	Atom	Angle/˚	Atom	Atom	Atom	Angle/˚
C2	S6	C7	98.30(8)	C15	C14	C11	122.29(15)
C2	N3	C4	105.70(14)	N3	C4	C21	120.98(15)
C5	N1	C11	128.37(14)	C5	C4	N3	110.07(15)
C2	N1	C5	106.17(13)	C5	C4	C21	128.94(16)
C2	N1	C11	125.18(14)	C18	C19	C14	120.36(16)
N1	C5	C20	125.28(14)	N3	C2	S6	124.17(12)
C4	C5	N1	105.99(14)	N3	C2	N1	112.05(14)

C4	C5	C20	128.70(15)	N1	C2	S6	123.73(12)
F8	C7	S6	112.98(12)	C14	C15	C16	120.14(16)
F9	C7	S6	114.07(12)	C16	C17	C18	119.29(16)
F9	C7	F8	106.30(14)	O13	C12	C11	108.95(13)
F10	C7	S6	107.99(11)	N1	C11	C14	110.95(13)
F10	C7	F8	107.44(14)	N1	C11	C12	109.83(12)
F10	C7	F9	107.75(14)	C14	C11	C12	114.94(14)
C19	C14	C11	118.63(15)	C19	C18	C17	120.52(17)
C15	C14	C19	119.07(15)	C17	C16	C15	120.61(19)

Table S7. Torsion Angles for **2g**.

A	B	C	D	Angle/˚	A	B	C	D	Angle/˚
O13	C12	C11	N1	-57.68(17)	C2	N3	C4	C5	0.21(18)
O13	C12	C11	C14	176.39(13)	C2	N3	C4	C21	-179.02(16)
N1	C5	C4	N3	-0.67(18)	C2	N1	C5	C4	0.84(17)
N1	C5	C4	C21	178.48(17)	C2	N1	C5	C20	-177.51(16)
C5	N1	C2	S6	176.83(12)	C2	N1	C11	C14	-139.34(15)
C5	N1	C2	N3	-0.76(18)	C2	N1	C11	C12	92.49(18)
C5	N1	C11	C14	47.5(2)	C15	C14	C19	C18	1.2(2)
C5	N1	C11	C12	-80.71(19)	C15	C14	C11	N1	-125.80(16)
C7	S6	C2	N3	-79.04(15)	C15	C14	C11	C12	-0.4(2)
C7	S6	C2	N1	103.65(14)	C20	C5	C4	N3	177.61(16)
C14	C19	C18	C17	-1.1(3)	C20	C5	C4	C21	-3.2(3)
C14	C15	C16	C17	-0.6(2)	C11	N1	C5	C4	175.06(15)
C4	N3	C2	S6	-177.24(12)	C11	N1	C5	C20	-3.3(3)
C4	N3	C2	N1	0.35(18)	C11	N1	C2	S6	2.4(2)
C19	C14	C15	C16	-0.4(2)	C11	N1	C2	N3	-175.22(14)
C19	C14	C11	N1	52.89(19)	C11	C14	C19	C18	-177.49(15)
C19	C14	C11	C12	178.25(14)	C11	C14	C15	C16	178.28(15)
C2	S6	C7	F8	-60.16(13)	C18	C17	C16	C15	0.8(3)
C2	S6	C7	F9	61.41(13)	C16	C17	C18	C19	0.0(3)
C2	S6	C7	F10	-178.84(12)					

Table S8. Hydrogen Atom Coordinates (Å×10^4) and Isotropic Displacement Parameters (Å^2×10^3) for **2g**.

Atom	*x*	*y*	*z*	U(eq)
H13	3146.03	2337.36	659.05	32
H19	8217.65	5007.87	4770.18	23
H15	6319.94	889.8	4460.14	24
H17	10493.77	1935.59	7493.97	28
H12A	5156.91	1505.35	2127.32	23
H12B	3909.15	1858.02	3088.3	23
H20A	9445.12	2384.22	3231.22	36

H20B	9961.88	2225.53	1827.03	36
H20C	8253.41	1454.77	2025.03	36
H11	5013.27	4260.12	3411.63	19
H18	10371	4324.96	6669.52	27
H21A	8941.62	4643.83	-1014.59	39
H21B	10186.58	3689.62	142.51	39
H21C	10273.78	5431.59	215.51	39
H16	8446.13	234.43	6398.57	28

5. References

1. (a) G. Mlostoń, T. Gendek, H. Heimgartner, *Helv. Chim. Acta* **1998**, *81*, 1585; (b) M. Jasiński, G. Mlostoń, P. Mucha, A. Linden, H. Heimgartner, *Helv. Chim. Acta* **2007**, 90, 1765; (c) M. Jasiński, G. Mlostoń, A. Linden, H. Heimgartner, *Helv. Chim. Acta* **2008**, *91*, 1916; (d) G. Mlostoń, M. Jasiński, *Arkivoc* **2011**, *vi*, 162; (e) G. Mlostoń, M. Jasiński, D. Rygielska, H. Heimgartner, *Heterocycles* **2011**, *83*, 765; (f) G. Mlostoń, M. Jasiński, H. Heimgartner, *Eur. J. Org. Chem.* **2011**, *13*, 2542; (g) M. Hossain, K. Pradhan, A. K. Nanda, *Tetrahedron Lett.* **2017**, *58*, 3772; (h) G. Mlostoń, M. Celeda, M. Jasiński, K. Urbaniak, P. J. Boratyński, P. R. Schreiner, H. Heimgartner, *Molecules* **2019**, *24*, 4398.
2. J. J. Worman, M. Shen, P. C. Nichols, *Can. J. Chem.* **1972**, *50*, 3923.
3. (a) O. V. Dolomanov, L. J. Bourhis, R. J Gildea, J. A. K. Howard, H. Puschmann, *J. Appl. Cryst.* **2009**, *42*, 339; (b) G. M. Sheldrick, *Acta Cryst.* A **2015**, *71*, 3; (c) G. M. Sheldrick, *Acta Cryst.* A **2008**, *64*, 112.
