# Supplementary figures and images for: One-Pot Telescoping S-Transfer and Trifluoromethylation for the Synthesis of 2-CF3S-Imidazoles with N-Oxides as Convenient Precursors
Source: J Org Chem. 2024 Sep 30;89(20):15331–5. doi: 10.1021/acs.joc.4c01761 (PMC11494641; doi:10.1021/acs.joc.4c01761)

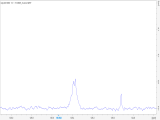

Supplement: Supplementary file 2 — jo4c01761_si_002.zip [file jo4c01761_si_002.zip › FID complete/2c/wkp-00142b [wkp-129] (13c)/10/pdata/1/thumb.png]
